# Perturbation analysis in finite LD‐QBD processes and applications to epidemic models

**DOI:** 10.1002/nla.2160

**Published:** 2018-03-05

**Authors:** A. Gómez‐Corral, M. López‐García

**Affiliations:** ^1^ Instituto de Ciencias Matemáticas CSIC‐UAM‐UC3M‐UCM Calle Nicolás Cabrera 13‐15 28049 Madrid Spain; ^2^ Department of Statistics and Operations Research School of Mathematics, Complutense University of Madrid 28040 Madrid Spain; ^3^ Department of Applied Mathematics, School of Mathematics University of Leeds LS2 9JT Leeds UK

**Keywords:** epidemic model, hitting time, matrix calculus, perturbation analysis, QBD process

## Abstract

In this paper, our interest is in the perturbation analysis of level‐dependent quasi‐birth‐and‐death (LD‐QBD) processes, which constitute a wide class of structured Markov chains. An LD‐QBD process has the special feature that its space of states can be structured by levels (groups of states), so that a tridiagonal‐by‐blocks structure is obtained for its infinitesimal generator. For these processes, a number of algorithmic procedures exist in the literature in order to compute several performance measures while exploiting the underlying matrix structure; among others, these measures are related to first‐passage times to a certain level L(0) and hitting probabilities at this level, the maximum level visited by the process before reaching states of level L(0), and the stationary distribution. For the case of a finite number of states, our aim here is to develop analogous algorithms to the ones analyzing these measures, for their perturbation analysis. This approach uses matrix calculus and exploits the specific structure of the infinitesimal generator, which allows us to obtain additional information during the perturbation analysis of the LD‐QBD process by dealing with specific matrices carrying probabilistic insights of the dynamics of the process. We illustrate the approach by means of applying multitype versions of the susceptible‐infective (SI) and susceptible‐infective‐susceptible (SIS) epidemic models to the spread of antibiotic‐sensitive and antibiotic‐resistant bacterial strains in a hospital ward.

## 1 INTRODUCTION

Recently, Caswell^1^ used matrix calculus to provide the sensitivities and elasticities (i.e., dimensionless sensitivities) of the dynamics of absorbing continuous‐time Markov chains (CTMCs) to arbitrary parameters, which may correspond to either the transition rates themselves or functions of these rates that have substantive meaning. More concretely, Caswell^1^ derived formulas for the sensitivity and elasticity of the moments of the time until absorption, the time spent in each transient state, and the number of visits to each transient state before absorption, being the resulting expressions applied to a model for the progress of colorectal cancer. The results in the work of Caswell^1^ are closely related to the investigation of absorbing Markov chains in discrete time, with applications to demographic and ecological problems in the work of Caswell.^2^

The reader is alerted to the fact that, although perturbation analysis of Markov chains is a long‐standing problem (see, for example, the survey by Avrachenkov et al.^3^), the approach suggested by Caswell^1^, ^2^ is not aligned with the classical treatment of the sensitivity analysis for perturbed Markov chains; see other works.^4^, ^5^, ^6^, ^7^, ^8^ In the classical framework, the problem is essentially based on bounding the distance between stationary distributions in a suitable functional space when the one‐step transition probability matrix **P** of a discrete‐time Markov chain is replaced by another one‐step transition probability matrix **P**′; see, for example, the papers by Li et al.^6^ and Seneta.^8^ For CTMCs, the impact on the steady‐state distribution is usually measured when the infinitesimal generator **Q** is *perturbed* to **Q**′(*ϵ*) = **Q** + *ϵ***Q**^∗^, where *ϵ* is a sufficiently small positive number and the *perturbation* matrix **Q** ^∗^ satisfies certain weak conditions; see, for example, section 3 in the work of Altman et al.^9^ The papers by Altman et al.,^9^ and Heidergott et al.^10^ are two representative contributions to the perturbation analysis for denumerable and finite Markov chains with applications to queueing models. In the epidemiological setting, Hautphenne et al.^11^ presented an analytical sensitivity treatment for a continuous‐time Markovian branching process and showed its applications to the early spread of an influenza‐like epidemic on a network of cities in the United States. Chitnis et al.^12^ computed sensitivity indices of the reproductive number and the endemic equilibrium point to the parameters on a deterministic model of malaria transmission. The interest of these authors^11^, ^12^ is in sensitivities/elasticities, instead of perturbations, with respect to a single parameter, whence the mathematical modelling in the works^11^, ^12^ may be seen as the *scalar* version of that in the works of Caswell.^1^, ^2^

In this paper, the aim is to complement the general treatment of Caswell^1^, ^2^ by taking advantage of the sparsity of the underlying matrices arising when analyzing a class of structured CTMCs. Specifically, the interest is in level‐dependent quasi‐birth‐and‐death (LD‐QBD) processes (see, e.g., section 7.2 in the work of Artalejo and Gómez‐Corral^13^), which are CTMCs in two dimensions, the *level* and the *phase*, such that the process only jumps across either adjacent levels or the same level in one transition. In analyzing LD‐QBD processes, matrix‐analytic methods^14^, ^15^ are popular as modeling tools that allow us to construct and study, under a unified and algorithmic tractable framework, a variety of stochastic models, such as epidemic models,^16^, ^17^, ^18^ inventory problems,^19^ reliability systems,^20^, ^21^ retrial queues,^22^, ^23^ and two‐species competition processes,^24^, ^25^, ^26^ among others. The starting point in our analysis is the paper by Gaver et al.,^27^ where the emphasis is upon obtaining numerical methods for evaluating stationary distributions and moments of first‐passage times in finite LD‐QBD processes. In the setting of perturbed QBD processes analyzed under the classical perspective, the paper by Li and Liu^28^ is an excellent work where censoring techniques and stochastic integral functionals are used to discuss infinitesimal sensitivity analysis, even in the case of perturbed structured Markov chains and Markovian arrival streams; for a related work, see the work of Dendievel et al.,^29^ where properties of the perturbed stationary distribution of a QBD process are derived by restricting the process to the first two levels of states.

The results to be presented here deal with perturbation analysis of finite LD‐QBD processes as an important tool for understanding how certain parameters—inherently linked to the dynamics of the model—determine the properties of the process, as well as for predicting how small changes in the environmental conditions will modify the outcome. We present an efficient computational approach to the perturbation analysis of finite LD‐QBD processes in terms of first‐passage times to a certain level *L*(0) and related hitting probabilities (Section 2.1), the maximum level visited by the process before reaching states in level *L*(0)(Section 2.2) and the stationary distribution (Section 2.3). Our results are motivated by, but not restricted to, epidemic models, whence we include two examples (Sections 3.1 and 3.2) describing the spread of two infectious agents among a finite population of individuals, under the assumption that each infectious agent confers immunity against the second agent. In the context of nosocomial pathogens, these epidemic models allow us to construct (Section 3.3) simple mathematical models of bacterial transmission within a hospital ward, in such a way that first‐passage times permit us to study the length of an outbreak; the stationary distribution is a long‐run description of the epidemic; and the maximum level visited by the LD‐QBD process is seen as an important measure in studying infectious peaks during an outbreak.

## 2 FINITE LD‐QBD PROCESSES

First, we introduce some terminology and notation for CTMCs and LD‐QBD processes. The interest is in a CTMC X={X(t):t≥0} on the two‐dimensional state space, as follows:

$$S=\left\{(i,j):\;0\leq i\leq K,\;0\leq j\leq M_{i}\right\},$$

where the first coordinate *i* represents the *level*, and the second one *j* is termed the *phase* of the state (*i*,*j*). We analyze the case of a potentially different but finite number *M*_*i*_+1 of phases per level, and we partition S as $\cup_{i=0}^{K}L(i)$ with *L*(*i*)={(*i*,*j*):0≤*j*≤*M*_*i*_} and 0≤*i*≤*K*. The CTMC X is then called an LD‐QBD process if transitions from a state are only permitted to either states in the same level or states in adjacent levels, and the infinitesimal rates are assumed to be potentially level dependent. By using a row‐to‐column orientation, this assumption yields a block‐tridiagonal infinitesimal generator

(1) $$Q=\left(\begin{array}{cccccc} Q_{0,0} & Q_{0,1} & & & & \\ Q_{1,0} & Q_{1,1} & Q_{1,2} & & & \\ & Q_{2,1} & Q_{2,2} & Q_{2,3} & & \\ & & \ddots & \ddots & \ddots & \\ & & & Q_{K-1,K-2} & Q_{K-1,K-1} & Q_{K-1,K}\\ & & & & Q_{K,K-1} & Q_{K,K} \end{array}\right),$$

where entries $q_{\left(i,j),(i^{\prime },j^{\prime }\right)}$ of $Q_{i,i^{\prime }}$ are linked to transitions from the state (*i*,*j*)∈*L*(*i*) to the state (*i*′, *j*′) ∈ *L*(*i*′), with *i*′ ∈ {*i* − 1, *i*, *i* + 1}; in writing Equation (1), we may assume a lexicographical ordering of states in S, which means that states in level *L*(*i*) precede those in *L*(*i*+1), and within level *L*(*i*) with 0≤*i*≤*K*, the state (*i*,*j*) precedes state (*i*,*j*+1) for phases 0≤*j*≤*M*_*i*_−1, so that ${\left(Q_{i,i^{\prime }}\right)}_{1+j,1+j^{\prime }}=q_{\left(i,j),(i^{\prime },j^{\prime }\right)}$. Note that, in the conservative case (i.e., $q_{\left(i,j),(i,j\right)}=-\sum_{\left(i^{\prime },j^{\prime })\in L^{*}(i,j\right)}q_{\left(i,j),(i^{\prime },j^{\prime }\right)}$, for any 0≤*i*≤*K* and 0≤*j*≤*M*_*i*_, with $L^{*}(i,j)=\cup_{i^{\prime }=i-1}^{i+1}L(i^{\prime })\backslash \{(i,j)\}$, and *L*(*i*′) is the empty set if *i*′ ∈ {−1, *K* + 1}), states of X are all positive recurrent as S is irreducible.

We shall consider here the general case where the entries of **Q** depend on some parameters in a column vector ***θ***=(*θ*_1_,*θ*_2_,…,*θ*_*s*_)^*T*^, in such a way that matrix **Q** remains the infinitesimal generator of a suitably defined conservative LD‐QBD process—on an irreducible class S of states—for sufficiently small perturbations of ***θ***. In Sections 2.1‐2.3, we present, in a unified way and algorithmically tractable manner, the perturbation analysis of three properties of the LD‐QBD process X with respect to ***θ***, and similar to the paper by Caswell,^1^ we use matrix calculus to differentiate matrices and vectors arising from the underlying algorithmic treatment of these properties. For the glossary of matrix notation and a summary of those matrix calculus properties used in Sections 2.1‐2.3, we refer the reader to Appendix A.

### 2.1 First‐passage times and hitting probabilities

Let *T*_(*i*, *j*)_ be the first‐passage time to level *L*(0), provided that the initial state of X is (*i*,*j*), and *p*_(*i*, *j*)_(*n*) be the probability, starting from (*i*,*j*), of reaching *L*(0) by visiting state (0,*n*), for phases 0≤*n*≤*M*_0_ and states (i,j)∈S. It is clear that, in the case *i*=0, first‐passage times verify *T*_(0, *j*)_=0 almost surely, and hitting probabilities are given by *p*_(0, *j*)_(*n*)=*δ*_*j*,*n*_, for phases 0≤*j*,*n*≤*M*_0_, where *δ*_*a*,*b*_ denotes Kronecker's delta.

In this section, we quantify the effects of changes in some entries of ***θ*** on the behavior of X in terms of *T*_(*i*, *j*)_ and *p*_(*i*, *j*)_(*n*) for states (i,j)∈S\L(0), which are closely related to an outbreak in epidemics (Section 3). The discussion that follows is based on the derivatives of the expectations $E\left[T_{\left(i,j\right)}^{l}\right]$ and probabilities *p*_(*i*, *j*)_(*n*) with respect to the parameter *θ*_*r*_, for integers *l*≥1 and 1≤*r*≤*s*. To this end, we first focus on first‐passage times *T*_(*i*, *j*)_ for states (i,j)∈S\L(0) and proceed in two steps; specifically, we present in the first step an algorithmic solution (Algorithm 1.A) for computing the moments $m_{\left(i,j\right)}^{\left(l\right)}=E\left[T_{\left(i,j\right)}^{l}\right]$, for integers *l*≥1, which is useful in deriving a second solution (Algorithm 1.B) for the partial derivatives $\partial E\left[T_{\left(i,j\right)}^{l}\right]/\partial \theta_{r}$, for integers *l*≥1 and 1≤*r*≤*s*.

To begin with, we condition on the first transition of the process X occurring from the initial state (i,j)∈S\L(0). Starting at (*i*,*j*), the first state visited by X may be any state (*i*′, *j*′) ∈ *L*^∗^(*i*, *j*) with probability $-q_{\left(i,j),(i,j\right)}^{-1}q_{\left(i,j),(i^{\prime },j^{\prime }\right)}$, and *T*_(*i*, *j*)_ can be readily decomposed into $T_{\left(i,j)\to (i^{\prime },j^{\prime }\right)}^{\prime }+T_{\left(i^{\prime },j^{\prime }\right)}$, where the random variable $T_{\left(i,j)\to (i^{\prime },j^{\prime }\right)}^{\prime }$ is exponentially distributed with parameter −*q*_(*i*, *j*),(*i*, *j*)_, and $T_{\left(i,j)\to (i^{\prime },j^{\prime }\right)}^{\prime }$ and $T_{\left(i^{\prime },j^{\prime }\right)}$ are independent by the Markovian property. This implies that, by conditioning on each possible first transition (*i*, *j*) → (*i*′, *j*′) of the process X, the Laplace–Stieltjes transforms $\varphi_{\left(i,j\right)}(z)=E[\exp\{-zT_{\left(i,j\right)}\}]$, for *R* *e*(*z*)≥0 and states (i,j)∈S\L(0), satisfy the equalities

(2) $$\varphi_{\left(i,j\right)}(z)=\frac{1}{z-q_{\left(i,j),(i,j\right)}}\left(\sum_{j^{\prime }=0}^{M_{i-1}}q_{\left(i,j),(i-1,j^{\prime }\right)}\varphi_{\left(i-1,j^{\prime }\right)}(z)+\sum_{j^{\prime }=0,j^{\prime }\neq j}^{M_{i}}q_{\left(i,j),(i,j^{\prime }\right)}\varphi_{\left(i,j^{\prime }\right)}(z)+(1-\delta_{i,K})\sum_{j^{\prime }=0}^{M_{i+1}}q_{\left(i,j),(i+1,j^{\prime }\right)}\varphi_{\left(i+1,j^{\prime }\right)}(z)\right),$$

with $\varphi_{\left(0,j^{\prime }\right)}(z)=1$ because $\varphi_{\left(i,j\right)}(z)=\sum_{\left(i^{\prime },j^{\prime })\in L^{*}(i,j\right)}-q_{\left(i,j),(i,j\right)}^{-1}q_{\left(i,j),(i^{\prime },j^{\prime }\right)}E[\exp\{-zT_{\left(i,j\right)}\}|(i,j)\to (i^{\prime },j^{\prime })]$ and $E[\exp\{-zT_{\left(i,j\right)}\}|(i,j)\to (i^{\prime },j^{\prime })]=E\left[\exp\left\{-zT_{\left(i,j)\to (i^{\prime },j^{\prime }\right)}^{\prime }\right\}\right]\varphi_{\left(i^{\prime },j^{\prime }\right)}(z)$ with $E\left[\exp\left\{-zT_{\left(i,j)\to (i^{\prime },j^{\prime }\right)}^{\prime }\right\}\right]=-{\left(z-q_{\left(i,j),(i,j\right)}\right)}^{-1}q_{\left(i,j),(i,j\right)}$. By multiplying Equation (2) by *z*−*q*_(*i*, *j*),(*i*, *j*)_, we derive the matrix equality

(3) $$z\varphi (z)=\bar{Q}\varphi (z)+b,$$

for the column vector *****φ*****(*z*) of Laplace–Stieltjes transforms *φ*_(*i*, *j*)_(*z*), for *R* *e*(*z*)≥0 and states (i,j)∈S\L(0), where the matrix $\bar{Q}$ is obtained from **Q** by deleting rows and columns associated with states of *L*(0); **b** is given by

$$b=\left(\begin{array}{c} Q_{1,0}\;1_{M_{0}+1}\\ 0_{J} \end{array}\right),$$

and $J=\sum_{i=2}^{K}M_{i}+K-1$ is the cardinality of the subset $\cup_{i=2}^{K}L(i)$. By taking derivatives in Equation (3) with respect to *z* at point *z*=0 and noting that $m_{\left(i,j\right)}^{\left(l\right)}={\left(-1\right)}^{l}d^{l}\varphi_{\left(i,j\right)}(z)/dz^{l}{|}_{z=0}$, we characterize the values $m_{\left(i,j\right)}^{\left(l\right)}$, for *l*≥1, as the solution to the system of linear equations

(4) $$m^{\left(l\right)}=Am^{\left(l\right)}+b^{\left(l\right)},$$

where

$$m^{\left(l\right)}=\left(\begin{array}{c} m_{1}^{\left(l\right)}\\ \vdots \\ m_{K}^{\left(l\right)} \end{array}\right),$$

with $m_{i}^{\left(l\right)}={\left(m_{\left(i,0\right)}^{\left(l\right)},\text{⋯},m_{\left(i,M_{i}\right)}^{\left(l\right)}\right)}^{T}$, for 1≤*i*≤*K*, and the matrix **A** has the structured form

(5) $$A=\left(\begin{array}{cccccc} A_{1,1} & A_{1,2} & & & & \\ A_{2,1} & A_{2,2} & A_{2,3} & & & \\ & A_{3,2} & A_{3,3} & A_{3,4} & & \\ & & \ddots & \ddots & \ddots & \\ & & & A_{K-1,K-2} & A_{K-1,K-1} & A_{K-1,K}\\ & & & & A_{K,K-1} & A_{K,K} \end{array}\right).$$

In Equation (5), the submatrix $A_{i,i^{\prime }}$ is obtained from $Q_{i,i^{\prime }}$ by dividing elements of its (1+*j*)th row, for integers 0≤*j*≤*M*_*i*_, by the value Δ_(*i*, *j*)_=−*q*_(*i*, *j*),(*i*, *j*)_, and the diagonal elements of **A**_*i*,*i*_ are all equal to zero, for 1≤*i*≤*K*, that is, the matrix **A** is related to the embedded jump chain and consists of one‐step transition probabilities from states of S\L(0) to states in S\L(0). The column vector **b** ^(*l*)^ in Equation (4) has the form

$$b^{\left(l\right)}=\left(\begin{array}{c} b_{1}^{\left(l\right)}\\ \vdots \\ b_{K}^{\left(l\right)} \end{array}\right),$$

where the subvector $b_{i}^{\left(l\right)}$ is specified by $lm_{i}^{\left(l-1\right)}/\Delta_{i}$ with $\Delta_{i}={\left(\Delta_{\left(i,0\right)},\text{⋯},\Delta_{\left(i,M_{i}\right)}\right)}^{T}$, for 1≤*i*≤*K*, which represents *element‐by‐element vector division*. Then, subvectors $m_{i}^{\left(l\right)}$ of moments in Equation (4) can be iteratively computed, starting with $m_{i}^{\left(0\right)}=1_{M_{i}+1}$, for 1≤*i*≤*K*, from previously computed subvectors $m_{i}^{\left(l-1\right)}$ of moments of order *l*−1, as indicated in Algorithm 1.A. The proof of Algorithm 1.A is based on a matrix version (see, e.g., p. 144 in the work of Ciarlet^30^) of the well‐known forward‐elimination–backward‐substitution method for solving a system of linear equations, and it is thus omitted.

Algorithm 1.A Computation of the expectations $m_{\left(i,j\right)}^{\left(l\right)}=E[T_{\left(i,j\right)}^{l}]$ of first‐passage times to level *L*(0), for *l*≥1 and states (i,j)∈S\L(0).

Step 1: Set *p*=0;
                    for *i*=1,…,*K*, evaluate
                                        
$\;m_{i}^{\left(p\right)}=1_{M_{i}+1}$;
                                        
$\;b_{i}^{\left(p+1\right)}=(p+1)\frac{m_{i}^{\left(p\right)}}{\Delta_{i}}$;
                    
$H_{K}=I_{M_{K}+1}-A_{K,K}$;
                    for *i*=*K*−1,…,1, evaluate
                                        
$\;H_{i}=I_{M_{i}+1}-A_{i,i}-A_{i,i+1}H_{i+1}^{-1}A_{i+1,i}$.
Step 2: While *p*<*l*, repeat
                                        *p*=*p*+1;
                                        
$\;J_{K}^{\left(p\right)}=b_{K}^{\left(p\right)}$;
                                        for *i*=*K*−1,…,1, evaluate
                                                  
$\;J_{i}^{\left(p\right)}=A_{i,i+1}H_{i+1}^{-1}J_{i+1}^{\left(p\right)}+b_{i}^{\left(p\right)}$;
                                        
$\;m_{1}^{\left(p\right)}=H_{1}^{-1}J_{1}^{\left(p\right)}$;
                                        for *i*=2,…,*K*, evaluate
                                                  
$\;m_{i}^{\left(p\right)}=H_{i}^{-1}(A_{i,i-1}m_{i-1}^{\left(p\right)}+J_{i}^{\left(p\right)})$;
                                        for *i*=1,…,*K*, evaluate
                                                     
$\;b_{i}^{\left(p+1\right)}=(p+1)\frac{m_{i}^{\left(p\right)}}{\Delta_{i}}$.

For every state (i,j)∈S\L(0), the random time *T*_(*i*,*j*)_ may be formulated as the time until absorption into an absorbing state 0 for a finite CTMC $X^{\prime }$ defined on the states {0}∪(S\L(0)), with initial probability vector $\left(0,{e^{T}}_{J^{\prime }}(f_{i,j})\right)$ and infinitesimal generator

$$Q^{\prime }=\left(\begin{array}{cc} 0\; & 0_{J^{\prime }}^{T}\\ q\; & \bar{Q} \end{array}\right),$$

where *J*′ = *M*_1_ + 1 + *J* is the cardinality of the class S\L(0) of transient states, the column vector **q** is given by $-\bar{Q}1_{J^{\prime }}$, $e_{J^{\prime }}(f_{i,j})$ is a column vector with *J* ^*′*^ entries that are all equal to zero, with the exception of a single one at the *f*_*i*, *j*_th entry, and $f_{i,j}=\sum_{i^{\prime }=1}^{i-1}M_{i^{\prime }}+i+j$, 1Under the assumption of a lexicographical labeling of states in S\L(0), the *f*_*i*,*j*_th entry of the vector $e_{J^{\prime }}(f_{i,j})$, with $f_{i,j}=\sum_{i^{\prime }=1}^{i-1}M_{i^{\prime }}+i+j$, amounts to the choice of (*i*,*j*) as the initial state of the process $X^{\prime }$. By section 2.3 in the work of Latouche and Ramaswami,^15^ this means that the first‐passage time *T*_(*i*, *j*)_ follows a *phase type* law with representation $\left({e^{T}}_{J^{\prime }}(f_{i,j}),\bar{Q}\right)$, and consequently, moments of *T*_(*i*, *j*)_ can be evaluated as

$$m_{\left(i,j\right)}^{\left(l\right)}=l!{e^{T}}_{J^{\prime }}(f_{i,j}){\left(-{\bar{Q}}^{-1}\right)}^{l}1_{J^{\prime }}.$$

Algorithm 1.A is then an alternative algorithmic solution to the general‐purpose expression $l!{e^{T}}_{J^{\prime }}(f_{i,j}){\left(-{\bar{Q}}^{-1}\right)}^{l}1_{J^{\prime }}$, where inverse matrices $H_{i}^{-1}$ of smaller orders *M*_*i*_+1 are progressively evaluated instead of a single inverse matrix of order *J* *′*.

Moreover, we stress that, because first‐passage times can be seen as absorption times, arguments by Caswell^1^ for sensitivity and elasticity of the moments of first‐passage times in absorbing CTMCs can be readily applied. Here, however, we suggest to adapt the matrix calculus approach of Caswell^1^, and by exploiting the block‐tridiagonal structure of **Q** in (1), we derive an analogous algorithm to Algorithm 1.A (Algorithm 1.B) allowing us to compute the partial derivatives of $E[T_{\left(i,j\right)}^{l}]$ in an efficient and unified manner. More concretely, Algorithm 1.B is derived by using matrix calculus to differentiate matrices and vectors in Algorithm 1.A with respect to the vector ***θ*** of parameters; for example, in evaluating $dm_{i}^{\left(p\right)}/d\theta^{T}$ from the equality $m_{i}^{\left(p\right)}=H_{i}^{-1}(A_{i,i-1}m_{i-1}^{\left(p\right)}+J_{i}^{\left(p\right)})$, for *p*<*l* and 2≤*i*≤*K*, in Algorithm 1.A (Step 2), straightforward algebra yields

$$\frac{dm_{i}^{\left(p\right)}}{d\theta^{T}}=-\left({\left(H_{i}^{-1}\left(A_{i,i-1}m_{i-1}^{\left(p\right)}+J_{i}^{\left(p\right)}\right)\right)}^{T}⊗H_{i}^{-1}\right)\frac{dvecH_{i}}{d\theta^{T}}+\left({\left(m_{i-1}^{\left(p\right)}\right)}^{T}⊗H_{i}^{-1}\right)\frac{dvecA_{i,i-1}}{d\theta^{T}}+H_{i}^{-1}A_{i,i-1}\frac{dm_{i-1}^{\left(p\right)}}{d\theta^{T}}+H_{i}^{-1}\frac{dJ_{i}^{\left(p\right)}}{d\theta^{T}},$$

as the reader may easily verify by applying properties 1–4 in Appendix A.

A particular feature of this approach is that it allows us to obtain some extra information about the effect of small perturbations in ***θ*** on the expected numbers of visits to states of *L*(*i*), before reaching states in *L*(*i*−1). To be concrete, we consider a family {X(i):0≤i≤K} of CTMCs, where X(i) with 1≤*i*≤*K* is the *restriction* of the process X, observed during those intervals of time spent at states in level *L*(*i*), before the process X moves *down* to level *L*(*i*−1) for the first time, and the process X(0) is the *restriction* of X observed at the lowest level *L*(0). This means that, for 1≤*i*≤*K*, X(i) is a transient CTMC. Then, for a fixed integer 1≤*i*≤*K*−1, the matrix $H_{i}^{-1}$ is related to the embedded jump chain of the restricted process X(i), and more particularly, its entry ${\left(H_{i}^{-1}\right)}_{1+j,1+j^{\prime }}$ can be seen as the expected number of visits to the state (*i*,*j* *′*), starting from the state (*i*,*j*), before the first visit of X(i) to any of the states in *L*(*i*−1) (or, equivalently, L(0)∪⋯∪L(i−1) because the process X(i) is forced to pass through states in level *L*(*i*−1) when it leaves level *L*(*i*)); in the special case *i*=*K*, the restricted version X(K) amounts to the original process X, and the entry ${\left(H_{K}^{-1}\right)}_{1+j,1+j^{\prime }}$ records the expected number of visits to the state (*K*,*j* *′*), starting from the state (*K*,*j*), before leaving level *L*(*K*). In analyzing the sensitivity of these expected numbers of visits to states in *L*(*i*), for integers 1≤*i*≤*K*, we remark here that, for integers 1≤*i*≤*K*, the derivatives of the matrix $H_{i}^{-1}$ can then be derived from the identity

$$\frac{dvecH_{i}^{-1}}{d\theta^{T}}=-\left({\left(H_{i}^{-1}\right)}^{T}⊗H_{i}^{-1}\right)\frac{dvecH_{i}}{d\theta^{T}},$$

where matrices $H_{i}^{-1}$ and $dvecH_{i}/d\theta^{T}$ are evaluated from Algorithms 1.A (Step 1) and 1.B (Step 1), respectively.

We note that, in Algorithm 1.B, matrices $dvecH_{i}^{-1}/d\theta^{T}$ are evaluated as a prerequisite for computing the submatrices $dm_{i}^{\left(l\right)}/d\theta^{T}$ containing the partial derivatives $\partial E[T_{\left(i,j\right)}^{l}]/\partial \theta_{r}$, for integers *l*≥1 and 1≤*r*≤*s* and states (i,j)∈S\L(0).

Algorithm 1.B Computation of the partial derivatives $\partial E[T_{\left(i,j\right)}^{l}]/\partial \theta_{r}$, for integers *l*≥1 and 1≤*r*≤*s* and states (i,j)∈S\L(0).

Step 1: Set *p*=0;
                    for *i*=1,…,*K*, evaluate
                                        
$\;\frac{dm_{i}^{\left(p\right)}}{d\theta^{T}}=0_{(M_{i}+1)\times s}$;
                                        
$\;\frac{db_{i}^{\left(p+1\right)}}{d\theta^{T}}=(p+1)\left(D^{-1}(\Delta_{i})\frac{dm_{i}^{\left(p\right)}}{d\theta^{T}}-\left(\left({\left(m_{i}^{\left(p\right)}\right)}^{T}D^{-1}(\Delta_{i})\right)⊗D^{-1}(\Delta_{i})\right)\right.\left.D(vecI_{M_{i}+1})(1_{M_{i}+1}⊗I_{M_{i}+1})\frac{d\Delta_{i}}{d\theta^{T}}\right)$;
                    
$\frac{dvecH_{K}}{d\theta^{T}}=-\frac{dvecA_{K,K}}{d\theta^{T}}$;
                    for *i*=*K*−1,…,1, evaluate
                                           
$\;\frac{dvecH_{i}}{d\theta^{T}}=-\frac{dvecA_{i,i}}{d\theta^{T}}-\left({\left(H_{i+1}^{-1}A_{i+1,i}\right)}^{T}⊗I_{M_{i+1}}\right)\frac{dvecA_{i,i+1}}{d\theta^{T}}$
                                                              
$\;+\left({\left(H_{i+1}^{-1}A_{i+1,i}\right)}^{T}⊗\left(A_{i,i+1}H_{i+1}^{-1}\right)\right)\frac{dvecH_{i+1}}{d\theta^{T}}-\left(I_{M_{i}+1}⊗\left(A_{i,i+1}H_{i+1}^{-1}\right)\right)\frac{dvecA_{i+1,i}}{d\theta^{T}}$.
Step 2: While *p*<*l*, repeat
                    *p*=*p*+1;
                    
$\;\frac{dJ_{K}^{\left(p\right)}}{d\theta^{T}}=\frac{db_{K}^{\left(p\right)}}{d\theta^{T}}$;
                    for *i*=*K*−1,…,1, evaluate
                                           
$\;\frac{dJ_{i}^{\left(p\right)}}{d\theta^{T}}\;=\;\left(\;{\left(H_{i+1}^{-1}J_{i+1}^{\left(p\right)}\right)}^{T}\;⊗\;I_{M_{i}+1}\right)\;\frac{dvecA_{i,i+1}}{d\theta^{T}}+A_{i,i+1}H_{i+1}^{-1}\frac{dJ_{i+1}^{\left(p\right)}}{d\theta^{T}}\;-\;\left(\;{\left(H_{i+1}^{-1}J_{i+1}^{\left(p\right)}\right)}^{T}\;⊗\;\left(A_{i,i+1}H_{i+1}^{-1}\right)\;\right)\;\frac{dvecH_{i+1}}{d\theta^{T}}\;+\;\frac{db_{i}^{\left(p\right)}}{d\theta^{T}}\;;$
                    
$\;\frac{dm_{1}^{\left(p\right)}}{d\theta^{T}}=-\left({\left(H_{1}^{-1}J_{1}^{\left(p\right)}\right)}^{T}⊗H_{1}^{-1}\right)\frac{dvecH_{1}}{d\theta^{T}}+H_{1}^{-1}\frac{dJ_{1}^{\left(p\right)}}{d\theta^{T}}$;
                    for *i*=2,…,*K*, evaluate
                                          
$\;\frac{dm_{i}^{\left(p\right)}}{d\theta^{T}}=-\left({\left(H_{i}^{-1}\left(A_{i,i-1}m_{i-1}^{\left(p\right)}+J_{i}^{\left(p\right)}\right)\right)}^{T}⊗H_{i}^{-1}\right)\frac{dvecH_{i}}{d\theta^{T}}$
                                                               
$\;+\left({\left(m_{i-1}^{\left(p\right)}\right)}^{T}⊗H_{i}^{-1}\right)\frac{dvecA_{i,i-1}}{d\theta^{T}}+H_{i}^{-1}A_{i,i-1}\frac{dm_{i-1}^{\left(p\right)}}{d\theta^{T}}+H_{i}^{-1}\frac{dJ_{i}^{\left(p\right)}}{d\theta^{T}}$;
                    for *i*=1,…,*K*, evaluate
                                           
$\;\frac{db_{i}^{\left(p+1\right)}}{d\theta^{T}}\;=\;(p+1)\;\left(\;D^{-1}(\Delta_{i})\frac{dm_{i}^{\left(p\right)}}{d\theta^{T}}-\left(\left({\left(m_{i}^{\left(p\right)}\right)}^{T}D^{-1}(\Delta_{i})\right)\;⊗\;D^{-1}(\Delta_{i})\right)\right.\;\;\left.\;D(vecI_{M_{i}+1})(1_{M_{i}+1}⊗I_{M_{i}+1})\frac{d\Delta_{i}}{d\theta^{T}}\right)\;.$

Similar to first‐passage times, the hitting probabilities *p*_(*i*, *j*)_(*n*), for states (i,j)∈S\L(0) and phases 0≤*n*≤*M*_0_, can be analyzed by using a first‐step argument, yielding

(6) $$p_{\left(i,j\right)}(n)=\frac{1}{-q_{\left(i,j),(i,j\right)}}\left(\sum_{j^{\prime }=0}^{M_{i-1}}q_{\left(i,j),(i-1,j^{\prime }\right)}p_{\left(i-1,j^{\prime }\right)}(n)+\sum_{j^{\prime }=0,j^{\prime }\neq j}^{M_{i}}q_{\left(i,j),(i,j^{\prime }\right)}p_{\left(i,j^{\prime }\right)}(n)+(1-\delta_{i,K})\sum_{j^{\prime }=0}^{M_{i+1}}q_{\left(i,j),(i+1,j^{\prime }\right)}p_{\left(i+1,j^{\prime }\right)}(n)\right).$$

Then, it is clear from Equations (2) and (6) that the hitting probabilities *p*_(*i*, *j*)_(*n*), for states (i,j)∈S\L(0) and phases 0≤*n*≤*M*_0_, can be determined as the solution to Equation (4) with the column vectors **m** ^(*l*)^ and **b** ^(*l*)^ replaced by

$$p(n)=\left(\begin{array}{c} p_{1}(n)\\ \vdots \\ p_{K}(n) \end{array}\right)\;\mathrm{and}\;\hat{b}(n)=\left(\begin{array}{c} {\hat{b}}_{1}(n)\\ \vdots \\ {\hat{b}}_{K}(n) \end{array}\right),$$

respectively, where $p_{i}(n)={\left(p_{\left(i,0\right)}(n),\text{⋯},p_{\left(i,M_{i}\right)}(n)\right)}^{T}$ and ${\hat{b}}_{i}(n)=\delta_{1,i}A_{i,i-1}e_{M_{i-1}+1}(n+1)$, for integers 1≤*i*≤*K*. Algorithm 2.A is then a simplified version of Algorithm 1.A, from which we may evaluate the hitting probability *p*_(*i*, *j*)_(*n*) as the (1+*j*)th entry of the column vector **p**_*i*_(*n*), for states (i,j)∈S\L(0) and phases 0≤*n*≤*M*_0_.

Algorithm 2.A Computation of the hitting probabilities *p*_(*i*,*j*)_(*n*), for states (i,j)∈S\L(0) and phases 0≤*n*≤*M*_0_+1.

    For *i*=*K*,…,1, evaluate
                    
$\;H_{i}=I_{M_{i}+1}-A_{i,i}-(1-\delta_{i,K})A_{i,i+1}H_{i+1}^{-1}A_{i+1,i}$;
                    
$\;{\hat{b}}_{i}(n)=\delta_{1,i}A_{i,i-1}e_{M_{i-1}+1}(n+1)$;
      
$\;J_{K}(n)={\hat{b}}_{K}(n)$;
      for *i*=*K*−1,…,1, evaluate
                    
$\;J_{i}(n)=A_{i,i+1}H_{i+1}^{-1}J_{i+1}(n)+{\hat{b}}_{i}(n)$;
      
$\;p_{1}(n)=H_{1}^{-1}J_{1}(n)$;
      for *i*=2,…,*K*, evaluate
                    
$\;p_{i}(n)=H_{i}^{-1}(A_{i,i-1}p_{i-1}(n)+J_{i}(n))$.

Regarding the perturbation of hitting probabilities, matrix calculus results (Appendix A) applied to Algorithm 2.A lead us to Algorithm 2.B with a solution for the partial derivatives *∂* *p*_(*i*, *j*)_(*n*)/*∂* *θ*_*r*_ for states (i,j)∈S\L(0), phases 0≤*n*≤*M*_0_, and integers 1≤*r*≤*s*, which are stored in the (1+*n*)th row and the *r*th column of the Jacobian matrix $dp_{i}(n)/d\theta^{T}$, for 1≤*i*≤*K*. Algorithm 2.B makes use of matrices $dvecH_{i}/d\theta^{T}$, previously computed in Algorithm 1.B.

Algorithm 2.B Computation of the partial derivatives *∂* *p*_(*i*, *j*)_(*n*)/*∂* *θ*_*r*_, for states (i,j)∈S\L(0), phases 0≤*n*≤*M*_0_, and integers 1≤*r*≤*s*.

For *i*=*K*,…,1, evaluate
                    
$\;\frac{d{\hat{b}}_{i}(n)}{d\theta^{T}}=\delta_{i,1}\left(e_{M_{i-1}+1}^{T}(n+1)⊗I_{M_{i}+1}\right)\frac{dvecA_{i,i-1}}{d\theta^{T}}$;
$\frac{dJ_{K}(n)}{d\theta^{T}}=\frac{d{\hat{b}}_{K}(n)}{d\theta^{T}}$;
for *i*=*K*−1,…,1, evaluate
                    
$\;\frac{dJ_{i}(n)}{d\theta^{T}}\;=\;\left(\;{\left(H_{i+1}^{-1}J_{i+1}(n)\right)}^{T}⊗I_{M_{i}+1}\right)\frac{dvecA_{i,i+1}}{d\theta^{T}}+A_{i,i+1}H_{i+1}^{-1}\frac{dJ_{i+1}(n)}{d\theta^{T}}-\left({\left(H_{i+1}^{-1}J_{i+1}(n)\right)}^{T}⊗\left(A_{i,i+1}H_{i+1}^{-1}\right)\right)\frac{dvecH_{i+1}}{d\theta^{T}}+\frac{d{\hat{b}}_{i}(n)}{d\theta^{T}};$
$\frac{dp_{1}(n)}{d\theta^{T}}=-\left({\left(H_{1}^{-1}J_{1}(n)\right)}^{T}⊗H_{1}^{-1}\right)\frac{dvecH_{1}}{d\theta^{T}}+H_{1}^{-1}\frac{dJ_{1}(n)}{d\theta^{T}}$;
for *i*=2,…,*K*, evaluate
                    
$\;\frac{dp_{i}(n)}{d\theta^{T}}\;=\;-\;\left(\;{\left(H_{i}^{-1}\left(\;A_{i,i-1}p_{i-1}(n)+J_{i}(n)\right)\;\right)\;}^{T}⊗H_{i}^{-1}\right)\;\frac{dvecH_{i}}{d\theta^{T}}\;+\;\left({\left(p_{i-1}(n)\right)}^{T}\;⊗\;H_{i}^{-1}\right)\frac{dvecA_{i,i-1}}{d\theta^{T}}\;+\;H_{i}^{-1}\left(A_{i,i-1}\frac{dp_{i-1}(n)}{d\theta^{T}}\;+\;\frac{dJ_{i}(n)}{d\theta^{T}}\right)\;.$

### 2.2 Maximum level visited before reaching level L(0)

In this section, we briefly present the perturbation analysis of the probability distribution of the maximum level $X_{\max}$ visited by the LD‐QBD process X before reaching states of level *L*(0), provided that *X*(0)=(*i*,*j*), for states (i,j)∈S\L(0). To this end, we first observe that the random variable $X_{\max}$ is identically distributed as its counterpart in the embedded jump chain. We then suggest to compute the conditional probabilities ${\tilde{p}}_{\left(i,j\right)}(x)=P(X_{\max}\geq x|X(0)=(i,j))$, for integers *x*∈{*i*+1,…,*K*} and initial states (i,j)∈S\L(0), by noting that ${\tilde{p}}_{\left(i,j\right)}(x)$ is equal to the probability that, starting from (*i*,*j*), the embedded jump process enters the subset $\cup_{i^{\prime }=x}^{K}L(i^{\prime })$ of states but avoiding states of *L*(0). Hence, for each fixed integer *x*∈{*i*+1,…,*K*}, we consider an absorbing discrete‐time process $\tilde{Y}(x)$ defined on the state space

$$\tilde{S}(x)=\{0\}\cup \overset{x-1}{\underset{i^{\prime }=1}{⋃}}\;L(i^{\prime })\cup \{x\},$$

where 0 and *x* are obtained by lumping the level *L*(0) and the subset $\cup_{i^{\prime }=x}^{K}L(i^{\prime })$ together to make two absorbing states. The one‐step transition probability matrix of $\tilde{Y}(x)$ has the structured form

$$\tilde{P}(x)=\left(\begin{array}{ccc} 1 & 0_{J^{\prime \prime \prime }}^{T} & 0\\ \tilde{a}(x) & \tilde{A}(x) & \tilde{b}(x)\\ 0 & 0_{J^{\prime \prime \prime }}^{T} & 1 \end{array}\right),$$

where we let $\tilde{a}(x)$ and $\tilde{b}(x)$ be $1_{J^{\prime \prime \prime }}-\tilde{A}(x)1_{J^{\prime \prime \prime }}-\tilde{b}(x)$ and $\left(\begin{array}{c} 0_{J^{\prime \prime }}\\ A_{x-1,x}1_{M_{x}+1} \end{array}\right)$, respectively, $J^{\prime \prime }=\sum_{i^{\prime }=1}^{x-2}M_{i^{\prime }}+x-2$, *J* ^*′′′*^=*M*_*x*−1_+1+*J* ^*′′*^, and

$$\tilde{A}(x)=\left(\begin{array}{ccccc} A_{1,1} & A_{1,2} & & & \\ A_{2,1} & A_{2,2} & A_{2,3} & & \\ & \ddots & \ddots & \ddots & \\ & & A_{x-2,x-3} & A_{x-2,x-2} & A_{x-2,x-1}\\ & & & A_{x-1,x-2} & A_{x-1,x-1} \end{array}\right).$$

In a similar manner to Equation (6), a first‐step argument allows us to observe that the conditional probabilities ${\tilde{p}}_{\left(i,j\right)}(x)=P(X_{\max}\geq x|X(0)=(i,j))$, for integers *x*∈{*i*+1,…,*K*} and initial states (i,j)∈S\L(0), can be thought of as *restricted* hitting probabilities verifying Equation (4) with obvious modifications in matrices and vectors. To be concrete, the one‐step transition probability matrix **A** in Equation (4) is replaced by $\tilde{A}(x)$, and the column vectors **m** ^(*l*)^ and **b** ^(*l*)^ are replaced, respectively, by $\tilde{p}(x)$ and $\tilde{b}(x)$, with

$$\tilde{p}(x)=\left(\begin{array}{c} {\tilde{p}}_{1}(x)\\ \vdots \\ {\tilde{p}}_{x-1}(x) \end{array}\right),$$

and ${\tilde{p}}_{i}(x)={\left({\tilde{p}}_{\left(i,0\right)}(x),\text{⋯}\;,{\tilde{p}}_{\left(i,M_{i}\right)}(x)\right)}^{T}$. This means that, for every integer *x*∈{*i*+1,…,*K*}, the conditional mass function of $X_{\max}$ can be derived from Algorithm 2.A as

$$P(X_{\max}=x|X(0)=(i,j))={\tilde{p}}_{\left(i,j\right)}(x)-(1-\delta_{x,K}){\tilde{p}}_{\left(i,j\right)}(x+1).$$

As a result, the partial derivatives $\partial P(X_{\max}=x|X(0)=(i,j))/\partial \theta_{r}$, for integers 1≤*r*≤*s*, are evaluated as $\partial {\tilde{p}}_{\left(i,j\right)}(x)/\partial \theta_{r}-(1-\delta_{x,K})\partial {\tilde{p}}_{\left(i,j\right)}(x+1)/\partial \theta_{r}$, whose terms are readily derived by adapting Algorithm 2.B in an appropriate manner.

### 2.3 Stationary regime

To determine the stationary distribution of the LD‐QBD process X, many approaches may be followed, and for general purposes, most of them yield algorithmic procedures. These algorithmic procedures are usually based on the tridiagonal‐by‐blocks form (1) of the infinitesimal generator **Q** without any further assumption, except that the LD‐QBD process X is irreducible. We focus here on the solution given by Gaver et al.,^27^ which proceeds in two steps: During the first step, the procedure progressively reduces the state space by removing one level at each iteration, until a CTMC defined on states of *L*(*K*) is constructed; once this CTMC on level *L*(*K*) is solved, the procedure iteratively computes the stationary vector of the process X in the second step, by adding back one level at each iteration. This yields the procedure described in Algorithm 3.A, which amounts to that in algorithm A in the work of Gaver et al.,^27^ from which we may evaluate the stationary probabilities $\pi_{\left(i,j\right)}=\lim_{t\to \infty }P(X(t)=(i,j)|X(0)=(i^{\prime },j^{\prime }))$ for states (i,j)∈S as the (1+*j*)th entry of the column vector **π**_*i*_, regardless of the initial state $(i^{\prime },j^{\prime })\in S$.

Algorithm 3.A (Linear level reduction algorithm; see section 2 in the work of Gaver et al.^27^) Computation of the stationary probabilities π_(*i*,*j*)_, for states (i,j)∈S.

Step 1: **B**_0_=**Q**_0,0_;
                    for *i*=1,…,*K*, evaluate
                                   
$\;B_{i}=Q_{i,i}+Q_{i,i-1}\left(-B_{i-1}^{-1}\right)Q_{i-1,i}$;
                    evaluate ${\tilde{\pi }}_{K}$ by solving
                                   
$\;B_{K}^{T}{\tilde{\pi }}_{K}=0_{M_{K}+1}$ with $1_{M_{K}+1}^{T}{\tilde{\pi }}_{K}=1$.
Step 2: Set $\bar{c}=1$;
                    for *i*=*K*−1,…,0, evaluate
                                   
$\;{\tilde{\pi }}_{i}={\left(-B_{i}^{-1}\right)}^{T}Q_{i+1,i}^{T}{\tilde{\pi }}_{i+1}$;
                                   
$\;\bar{c}=\bar{c}+1_{M_{i}+1}^{T}{\tilde{\pi }}_{i}$;
                    for *i*=0,…,*K*, evaluate
                                   
$\;\pi_{i}={\bar{c}}^{-1}{\tilde{\pi }}_{i}$.

Then, an appeal to the matrix calculus results in Appendix A allows us to differentiate matrices and vectors with respect to the vector ***θ*** of parameters on Steps 1–2 of Algorithm 3.A and, consequently, to provide the perturbation analysis of X in terms of partial derivatives of the stationary probabilities *∂*π_(*i*,*j*)_/*∂* *θ*_*r*_, for integers 1≤*r*≤*s*, which are located at the (1+*j*)th row and the *r*th column of the matrix $d\pi_{i}/d\theta^{T}$, for states (i,j)∈S.

Algorithm 3.B Computation of the partial derivatives *∂*π_(*i*,*j*)_/*∂* *θ*_*r*_, for integers 1≤*r*≤*s* and states (i,j)∈S.

Step 1: $\frac{dvecB_{0}}{d\theta^{T}}=\frac{dvecQ_{0,0}}{d\theta^{T}}$;
                    
$\;\frac{dvecB_{0}^{T}}{d\theta^{T}}=\frac{dvecQ_{0,0}^{T}}{d\theta^{T}}$;
                    for *i*=1,…,*K*, evaluate
                                            
$\;\frac{dvecB_{i}}{d\theta^{T}}=\frac{dvecQ_{i,i}}{d\theta^{T}}-\left({\left(B_{i-1}^{-1}Q_{i-1,i}\right)}^{T}⊗I_{M_{i}+1}\right)\frac{dvecQ_{i,i-1}}{d\theta^{T}}$
                                                            
$\;+\left({\left(B_{i-1}^{-1}Q_{i-1,i}\right)}^{T}⊗\left(Q_{i,i-1}B_{i-1}^{-1}\right)\right)\frac{dvecB_{i-1}}{d\theta^{T}}-\left(I_{M_{i}+1}⊗\left(Q_{i,i-1}B_{i-1}^{-1}\right)\right)\frac{dvecQ_{i-1,i}}{d\theta^{T}}$;
                                            
$\;\frac{dvecB_{i}^{T}}{d\theta^{T}}=\frac{dvecQ_{i,i}^{T}}{d\theta^{T}}-\left(\left(Q_{i,i-1}B_{i-1}^{-1}\right)⊗I_{M_{i}+1}\right)\frac{dvecQ_{i-1,i}^{T}}{d\theta^{T}}$
                                                            
$\;+\left(\left(Q_{i,i-1}B_{i-1}^{-1}\right)⊗{\left(B_{i-1}^{-1}Q_{i-1,i}\right)}^{T}\right)\frac{dvecB_{i-1}^{T}}{d\theta^{T}}-\left(I_{M_{i}+1}⊗{\left(B_{i-1}^{-1}Q_{i-1,i}\right)}^{T}\right)\frac{dvecQ_{i,i-1}^{T}}{d\theta^{T}}$;
                    evaluate $d{\tilde{\pi }}_{K}/d\theta^{T}$ by solving
                                              
$\;\left({\tilde{\pi }}_{K}^{T}⊗I_{M_{K}+1}\right)\frac{dvecB_{K}^{T}}{d\theta^{T}}=-B_{K}^{T}\frac{d{\tilde{\pi }}_{K}}{d\theta^{T}}$ with $1_{M_{K}+1}^{T}\frac{d{\tilde{\pi }}_{K}}{d\theta^{T}}=0_{s}^{T}$.
Step 2: Set $\frac{d\bar{c}}{d\theta^{T}}=0_{s}^{T}$;
                    for *i*=*K*−1,…,0, evaluate
                                            
$\;\frac{d{\tilde{\pi }}_{i}}{d\theta^{T}}=\left(\left({\tilde{\pi }}_{i+1}^{T}Q_{i+1,i}B_{i}^{-1}\right)⊗{\left(B_{i}^{-1}\right)}^{T}\right)\frac{dvecB_{i}^{T}}{d\theta^{T}}-\left({\tilde{\pi }}_{i+1}^{T}⊗{\left(B_{i}^{-1}\right)}^{T}\right)\frac{dvecQ_{i+1,i}^{T}}{d\theta^{T}}-{\left(Q_{i+1,i}B_{i}^{-1}\right)}^{T}\frac{d{\tilde{\pi }}_{i+1}}{d\theta^{T}}$;
                                            
$\;\frac{d\bar{c}}{d\theta^{T}}=\frac{d\bar{c}}{d\theta^{T}}+1_{M_{i}+1}^{T}\frac{d{\tilde{\pi }}_{i}}{d\theta^{T}}$;
                    for *i*=0,…,*K*, evaluate
                                            
$\;\frac{d\pi_{i}}{d\theta^{T}}={\bar{c}}^{-1}\left(\frac{d{\tilde{\pi }}_{i}}{d\theta^{T}}-\pi_{i}\frac{d\bar{c}}{d\theta^{T}}\right)$.

For a fixed integer 0≤*i*≤*K*−1, the matrix **B**_*i*_ in Algorithm 3.A (Step 1) can be thought of as the infinitesimal generator of the *restriction* $\bar{X}(i)$ of the LD‐QBD process X, observed during those intervals of time spent at level *L*(*i*), before it enters level *L*(*i*+1) for the first time. It is clear that the state space of the restriction $\bar{X}(i)$ is given by *L*(*i*), and $\bar{X}(i)$ is a transient CTMC in the case 0≤*i*≤*K*−1, whereas the restriction $\bar{X}(K)$ of the process X to states in *L*(*K*) is positive recurrent. Therefore, the entry ${\left(-B_{i}^{-1}\right)}_{1+j,1+j^{\prime }}$ is interpreted as recording the expected total time spent in the state (*i*,*j* *′*), starting from the state (*i*,*j*), before the first visit of $\bar{X}(i)$ to any of the states in *L*(*i*+1), for integers 1≤*i*≤*K*−1. ${\left(-B_{0}^{-1}\right)}_{1+j,1+j^{\prime }}$ represents the expected total time spent in (0,*j* *′*), given that the process X starts from the state (0,*j*), before leaving level *L*(0). Thus, regarding the effect of small perturbations in ***θ*** on these expected total times spent at states in *L*(*i*) with 0≤*i*≤*K*−1, we point out here that, for phases 0≤*j*,*j* *′*≤*M*_*i*_ and integers 1≤*r*≤*s*, the partial derivatives $\partial {\left(-B_{i}^{-1}\right)}_{1+j,1+j^{\prime }}/\partial \theta_{r}$ can be readily derived from the matrices $-B_{i}^{-1}$ (Step 1 in Algorithm 3.A) and $dvec(-B_{i})/d\theta^{T}$ (Step 1 in Algorithm 3.B), because $dvec(-B_{i}^{-1})/d\theta^{T}=-({\left(-B_{i}^{-1}\right)}^{T}⊗(-B_{i}^{-1}))dvec(-B_{i})/d\theta^{T}$.

## 3 APPLICATIONS TO EPIDEMIC MODELS

LD‐QBD processes are natural tools for the analysis of epidemic models governed by exponential laws. In Sections 3.1 and 3.2, we briefly discuss two models with epidemics in competition; specifically, two epidemic agents are assumed to be simultaneously present in a finite population, in such a way that they interact to increase or decrease each other's effectiveness, and each agent confers immunity against the other epidemic agent. In Section 3.3, these models are linked to the work by Lipsitch et al.,^31^ where a mathematical model of bacterial transmission within a hospital is described in order to study the effects of measures to control nosocomial transmission of bacteria and reduce antimicrobial resistance in nosocomial pathogens.

### 3.1 The multitype S I epidemic model

**Figure 1:**
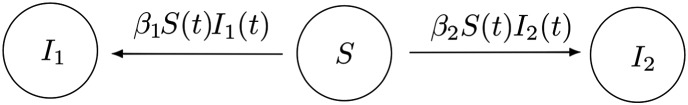
Multitype S I stochastic epidemic model

The first example corresponds to the *S* *I*_1_,*I*_2_ epidemic model analyzed by Saunders^32^ (see also Billard et al.^33^), which describes the spread of two types of infectious diseases—termed *type‐1* and *type‐2*—among a homogeneously mixed closed population of *N* individuals. Infected individuals do not recover, but suffering one type of infectious disease provides immunity against the other. By denoting by *S*(*t*), *I*_1_(*t*), and *I*_2_(*t*), the number of susceptible individuals, and the numbers of type‐1 and type‐2 infectives, respectively, at time t≥0, we may describe the dynamics of the *S* *I*_1_,*I*_2_ epidemic model in terms of the finite CTMC X={(S(t),J(t)):t≥0}, where *S*(*t*)=*N*−*I*_1_(*t*)−*I*_2_(*t*) and *J*(*t*)=*I*_1_(*t*)−*I*_1_ with initial numbers *I*_1_(0)=*I*_1_ and *I*_2_(0)=*I*_2_ of infectives. The state space of X has the form

$$S=\left\{(i,j)\in N_{0}\times N_{0}:0\leq i\leq N-I_{1}-I_{2},0\leq j\leq N-I_{1}-I_{2}-i\right\},$$

and transitions among states are due to either a new type‐1 infection (i.e., (*i*,*j*)→(*i*−1,*j*+1)) with rate *i*(*j*+*I*_1_)*β*_1_ or a new type‐2 infection (i.e., (*i*,*j*)→(*i*−1,*j*)) with rate *i*(*N*−*i*−*j*−*I*_1_)*β*_2_, for strictly positive contact rates *β*_1_ and *β*_2_; see Figure 1.

Let *T* be the time to reach the end of the epidemic spread, that is, T=inf{t≥0:S(t)=0}. In order to analyze the numbers *I*_1_(*T*) and *I*_2_(*T*) of infectives at time *T*, we may decompose the state space S by levels as $\cup_{i=0}^{N-I_{1}-I_{2}}L(i)$ with $L(i)=\{(i,j)\in S:0\leq j\leq N-I_{1}-I_{2}-i\}$, whence *K*=*N*−*I*_1_−*I*_2_ and level *L*(*i*) consists of *M*_*i*_+1=*N*−*I*_1_−*I*_2_−*i*+1 states, according to notation in Section 2. This labeling of states leads us to an LD‐QBD process defined on S, whose infinitesimal generator is specified according to Equation (1) as follows:

1. For integers 1≤*i*≤*N*−*I*_1_−*I*_2_, the (1+*j*,1+*j* *′*)th entry of **Q**_*i*,*i*−1_ is given by
  $${\left(Q_{i,i-1}\right)}_{1+j,1+j^{\prime }}=\left\{\begin{array}{ll} i(j+I_{1})\beta_{1},\; & \mathrm{if}\;j^{\prime }=j+1,\\ i(N-i-j-I_{1})\beta_{2},\; & \mathrm{if}\;j^{\prime }=j,\\ 0,\; & \text{otherwise}, \end{array}\right.$$
  for 0≤*j*≤*M*_*i*_ and 0≤*j* ^*′*^≤*M*_*i*−1_.
2. The submatrix **Q**_0,0_ takes the form $Q_{0,0}=0_{\left(M_{0}+1)\times (M_{0}+1\right)}$ and, for integers 1≤*i*≤*N*−*I*_1_−*I*_2_, the submatrix **Q**_*i*,*i*_ is a diagonal matrix with diagonal entries (**Q**_*i*,*i*_)_1+*j*,1+*j*_=−(( *j*+*I*_1_)*i* *β*_1_+(*N*−*i*−*j*−*I*_1_)*i* *β*_2_), for 0≤*j*≤*M*_*i*_.
3. For integers 0≤*i*≤*N*−*I*_1_−*I*_2_−1, the submatrix **Q**_*i*,*i*+1_ is a null matrix, that is, $Q_{i,i+1}=0_{\left(M_{i}+1)\times (M_{i+1}+1\right)}$.

In the terminology of Sections 2.1‐2.3, the random time *T* corresponds to the time instant $T_{\left(N-I_{1}-I_{2},0\right)}$ to reach states of *L*(0), provided that X starts from state (*N*−*I*_1_−*I*_2_,0). Thus, the joint probability distribution of the numbers (*I*_1_(*T*),*I*_2_(*T*)) of type‐1 and type‐2 infectives at the end of the epidemic spread amounts to the hitting probabilities $p_{\left(N-I_{1}-I_{2},0\right)}(i_{1}-I_{1})$, for integers *I*_1_≤*i*_1_≤*N*−*I*_2_, because the event {*I*_1_(*T*)=*i*_1_,*I*_2_(*T*)=*N*−*i*_1_} is equivalent to {*S*(*T*)=0,*J*(*T*)=*i*_1_−*I*_1_}.

### 3.2 The multitype S I S epidemic model

**Figure 2:**
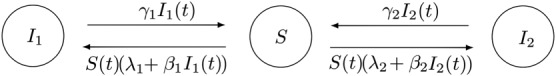
Multitype S I S stochastic epidemic model

In this section, we focus on a multitype *S* *I* *S* epidemic model with two strains and external sources of infection, which is shown in Figure 2. Similar to the case analyzed in Section 3.1, the resulting *S* *I*_1_,*I*_2_ *S* epidemic model describes the spread of two types of infection among a closed population of *N* homogeneous individuals, where now a susceptible individual can become a type‐*k* infective, for *k*∈{1,2}, due to either external factors (with rate *λ*_*k*_≥0) or an infectious contact with a type‐*k* infective (with rate *β*_*k*_>0). In the spirit of the work of Kirupaharan and Allen,^34^ any type‐*k* infective cannot be infected by any type‐*k* *′* infective, with *k* *′*≠*k*, until his/her infectious period expires. Type‐*k* infectious periods are assumed to be exponentially distributed with mean $\gamma_{k}^{-1}$, for *k*∈{1,2}.

The *S* *I*_1_,*I*_2_ *S* epidemic process is analyzed by using the random variables *S*(*t*), *I*_1_(*t*), and *I*_2_(*t*) at an arbitrary time *t*, with *S*(0)=*N*−*I*_1_−*I*_2_, *I*_1_(0)=*I*_1_, *I*_2_(0)=*I*_2_ representing the initial conditions. Nevertheless, the construction of the underlying LD‐QBD process in Sections 3.2.1 and 3.2.2 shall depend on the probabilistic descriptors under study, which are related to the *global* outbreak (Section 3.2.1), the outbreak corresponding to the *k*th strain (Section 3.2.2), and the stationary distribution (Section 3.2.3).

#### 3.2.1 Descriptors of the global outbreak

Let *T* be the time for both strains to become extinct for the first time and $X_{\max}$ be the global peak of infection during the outbreak, that is, $T=\inf\{t\geq 0:I_{1}(t)+I_{2}(t)=0\}$ reflects the length of a global outbreak, and $X_{\max}=\max\{I_{1}(t)+I_{2}(t):0\leq t<T\}$ records the maximum number of simultaneously infected individuals before the global outbreak expires. Because these random indexes refer to *I*_1_(*t*)+*I*_2_(*t*), we define here the process $X_{1}$ as

$$X_{1}=\{(I(t),J(t)):t\geq 0\},$$

where *I*(*t*)=*I*_1_(*t*)+*I*_2_(*t*) and *J*(*t*)=*I*_2_(*t*), so that the number of susceptible individuals, and the numbers of type‐1 and type‐2 infectives are given by *S*(*t*)=*N*−*I*(*t*), *I*_1_(*t*)=*I*(*t*)−*J*(*t*), and *I*_2_(*t*)=*J*(*t*), respectively. The process $X_{1}$ can be seen as an LD‐QBD process defined on the state space $S_{1}=\cup_{i=0}^{N}L_{1}(i)$ with *i*th level *L*_1_(*i*)={(*i*,*j*):0≤*j*≤*i*}, for integers 0≤*i*≤*N*, that is, *K*=*N* and level *L*_1_(*i*) contains *M*_*i*_+1=*i*+1 states, for 0≤*i*≤*N*. This means that, under the initial conditions *I*_1_(0)=*I*_1_ and *I*_2_(0)=*I*_2_, the random time *T* corresponds to the first‐passage time $T_{\left(I_{1}+I_{2},I_{2}\right)}$, and the probability distribution of $X_{\max}$ can be determined by analyzing the distribution of the maximum level visited by the process $X_{1}$ before reaching states of level *L*_1_(0). Therefore, moments of *T* are progressively computed from Algorithm 1.A, whereas their local sensitivity analysis is carried out by means of Algorithm 1.B; in a similar manner, the conditional probabilities $P(X_{\max}=x|(I(0),J(0))=(I_{1}+I_{2},I_{2}))$ and their corresponding derivatives are derived from an adaptation of Algorithms 2.A and 2.B (according to our comments in Section 2.2). The submatrices $Q_{i,i^{\prime }}$, for *i* *′*∈{*i*−1,*i*,*i*+1}, to be used in these algorithms are specified by the following:

1. For 1≤*i*≤*N*, the (1+*j*,1+*j* *′*)th entry of **Q**_*i*,*i*−1_ is given by
  $${\left(Q_{i,i-1}\right)}_{1+j,1+j^{\prime }}=\left\{\begin{array}{ll} j\gamma_{2},\; & \text{if}\;j^{\prime }=j-1,\\ (i-j)\gamma_{1},\; & \text{if}\;j^{\prime }=j,\\ 0,\; & \text{otherwise}, \end{array}\right.$$
  for 0≤*j*≤*i* and 0≤*j* *′*≤*i*−1.
2. For 0≤*i*≤*N*, the submatrix **Q**_*i*,*i*_ has diagonal form and its diagonal entries are given by (**Q**_*i*,*i*_)_1+*j*,1+*j*_=−((*N*−*i*)(*λ*_1_+*λ*_2_+(*i*−*j*)*β*_1_+*j* *β*_2_)+(*i*−*j*)*γ*_1_+*j* *γ*_2_), for 0≤*j*≤*i*.
3. For 0≤*i*≤*N*−1, the (1+*j*,1+*j* *′*)th entry of **Q**_*i*,*i*+1_ is given by
  $${\left(Q_{i,i+1}\right)}_{1+j,1+j^{\prime }}=\left\{\begin{array}{ll} (N-i)(\lambda_{1}+(i-j)\beta_{1}),\; & \text{if}\;j^{\prime }=j,\\ (N-i)(\lambda_{2}+j\beta_{2}),\; & \text{if}\;j^{\prime }=j+1,\\ 0,\; & \text{otherwise}, \end{array}\right.$$
  for 0≤*j*≤*i* and 0≤*j* *′*≤*i*+1.

#### 3.2.2 Descriptors of the outbreak for the type‐k strain

For *k*∈{1,2}, we let *T*(*k*) denote the time before extinction of type‐*k* infectives for the first time (i.e., $T(k)=\inf\{t\geq 0:I_{k}(t)=0\}$), $I_{k^{\prime }}(T(k))$ be the number of type‐*k* *′* infectives (with *k* *′*≠*k*) when this occurs, and $X_{\max}(k)$ represent the peak of infection for strain *k* during the time interval [0,*T*(*k*)) (i.e., $X_{\max}(k)=\max\{I_{k}(t):0\leq t<T(k)\}$). Without any loss of generality, we focus on the case *k*=1 and define the CTMC $X_{2}=\{(I_{1}(t),I_{2}(t)):t\geq 0\}$ on the states of $S_{2}=\{(i,j):0\leq i\leq N,0\leq j\leq N-i\}$. By decomposing the state space $S_{2}$ by levels $\cup_{i=0}^{N}L_{2}(i)$ with *L*_2_(*i*)={(*i*,*j*):0≤*j*≤*N*−*i*} (i.e., *K*=*N* and *L*_2_(*i*) contains *M*_*i*_+1=*N*−*i*+1 states), we may formulate $X_{2}$ as an LD‐QBD process with the following submatrices $Q_{i,i^{\prime }}$, for *i* *′*∈{*i*−1,*i*,*i*+1}:

1. For 1≤*i*≤*N*, the non‐null entries of **Q**_*i*,*i*−1_ are given by (**Q**_*i*,*i*−1_)_1+*j*,1+*j*_=*i* *γ*_1_, for 0≤*j*≤*N*−*i*.
2. For 0≤*i*≤*N*, the entries of **Q**_*i*,*i*_ are given by
  $${\left(Q_{i,i}\right)}_{1+j,1+j^{\prime }}=\left\{\begin{array}{ll} j\gamma_{2},\; & \text{if}\;j^{\prime }=j-1,\\ -((N-i-j)(\lambda_{1}+\lambda_{2}+i\beta_{1}+j\beta_{2})+i\gamma_{1}+j\gamma_{2}),\; & \text{if}\;j^{\prime }=j,\\ (N-i-j)(\lambda_{2}+j\beta_{2}),\; & \text{if}\;j^{\prime }=j+1,\\ 0,\; & \text{otherwise}, \end{array}\right.$$
  for 0≤*j*≤*N*−*i* and 0≤*j* *′*≤*N*−*i*.
3. For 0≤*i*≤*N*−1, the non‐null entries of **Q**_*i*,*i*+1_ are given by (**Q**_*i*,*i*+1_)_1+*j*,1+*j*_=(*N*−*i*−*j*)(*λ*_1_+*i* *β*_1_), for 0≤*j*≤*N*−*i*−1.

#### 3.2.3 Stationary measures

Unlike the descriptors in Sections 3.2.1 and 3.2.2, which can be analyzed for nonnegative external infection rates *λ*_1_,*λ*_2_≥0, the analysis of the stationary distribution needs the assumption of at least an external infection stream (i.e., *λ*_1_>0 and /or *λ*_2_>0), so that the CTMC under analysis is positive recurrent. Then, the stationary distribution and its perturbation analysis can be readily evaluated by applying Algorithms 3.A and 3.B to either the process $X_{1}$ or the process $X_{2}$, because they only differ in the underlying labeling of states. Once the stationary distribution is in hand, it is possible to compute the mean and standard deviation of the numbers *I*_1_(*∞*) and *I*_2_(*∞*) of type‐1 and type‐2 infectives, respectively, in the long term.

### 3.3 A mathematical model of bacterial transmission

We link the *S* *I*_1_,*I*_2_, and *S* *I*_1_,*I*_2_ *S* epidemic models to the deterministic model in figure A in the work of Lipsitch et al.^31^ for the spread of two bacterial strains in a hospital ward. Lipsitch et al.^31^ considered an antibiotic‐sensitive (AS) bacterial strain and an antibiotic‐resistant (AR) bacterial strain, termed *strain 1* and *strain 2*, respectively, spreading among patients, such that the infection by one bacterial strain provides immunity against the other. Because antibiotics are commonly used in hospitals to prevent a wide range of conditions, Lipsitch et al.^31^ assumed that patients in the ward are routinely provided *antibiotics 1* and *2*, regardless of these patients being infected or not by bacteria; more concretely, antibiotic 1 is only effective against the AS bacterial strain, whereas antibiotic 2 is effective against both strains of bacteria. The acquisition of resistance by bacteria can lead to some *fitness cost*, amounting to a reduction of the bacterial strain infectiousness due to the corresponding mutation; to represent this fact, Lipsitch et al.^31^ considered a common infection rate *β*=1.0 days^−1^ and set *β*_1_=*β* and *β*_2_=(1−*c*)*β* with *c*∈(0,1). Spontaneous clearance of sensitive and resistant bacteria occurs at a rate *γ*, and contributions of antibiotics 1 and 2 to this recovery are represented by rates *τ*_1_ and *τ*_2_. Patients are assumed to be admitted by and discharged from the hospital ward at a common rate *μ*.

In our numerical experiments (Tables 1, 2, 3, 4), we consider a hospital ward with *N*=20 patients, initial numbers (*I*_1_,*I*_2_)=(1,1) of infectives, and values *c*∈{0.05,0.1,0.25} of fitness cost. It should be pointed out that, unlike the paper of Lipsitch et al.,^31^ where the deterministic model is related to frequencies, we shall consider from now on rates *β*_1_=*N* ^−1^ *β* and *β*_2_=*N* ^−1^(1−*c*)*β*, because the random variables in the underlying LD‐QBD processes X (Section 3.1), and $X_{1}$ and $X_{2}$(Section 3.2) amount to numbers of infectives.

**Table 1:** Means and standard deviations of the time T until the end of the epidemic spread and of the numbers I_1_(T) and I_2_(T) of type‐1 and type‐2 infectives when this occurs in the S I_1_,I_2_ epidemic model

Descriptor	***c* = 0.05**	***c* = 0.1**	***c* = 0.25**
*E*[*T*]	6.19405	6.34476	6.77960
*σ*(*T*)	1.71320	1.75954	1.91860
*E*[*I*_1_(*T*)]	10.43524	10.89166	12.38137
*σ*(*I*_1_(*T*))	5.46754	5.43652	5.18336
*E*[*I*_2_(*T*)]	9.56475	9.10833	7.61862
*σ*(*I*_2_(*T*))	5.46754	5.43652	5.18336

Note. (Strain 1: antibiotic sensitive; strain 2: antibiotic resistant.)

**Table 2:** Elasticities of the descriptors in Table 1 with respect to various parameters in the S I_1_,I_2_ epidemic model

c	Elasticities	***θ* = *β*_1_**	***θ* = *β*_2_**	***θ* = *c***	***θ* = *β***
0.05	$\frac{\partial E[T]/\partial \theta }{\theta^{-1}E[T]}$	−0.53632	−0.46367	−0.00227	−1
	$\frac{\partial \sigma (T)/\partial \theta }{\theta^{-1}\sigma (T)}$	−0.50385	−0.49614	+0.02611	−1
	$\frac{\partial E[I_{1}(T)]/\partial \theta }{\theta^{-1}E[I_{1}(T)]}$	+0.81180	−0.81180	+0.04272	0
	$\frac{\partial \sigma (I_{1}(T))/\partial \theta }{\theta^{-1}\sigma (I_{1}(T))}$	−0.06897	+0.06897	−0.00363	0
	$\frac{\partial E[I_{2}(T)]/\partial \theta }{\theta^{-1}E[I_{2}(T)]}$	−0.88569	+0.88569	−0.04661	0
	$\frac{\partial \sigma (I_{2}(T))/\partial \theta }{\theta^{-1}\sigma (I_{2}(T))}$	−0.06897	+0.06897	−0.00363	0
0.10	$\frac{\partial E[T]/\partial \theta }{\theta^{-1}E[T]}$	−0.57432	−0.42567	−0.00411	−1
	$\frac{\partial \sigma (T)/\partial \theta }{\theta^{-1}\sigma (T)}$	−0.50908	−0.49091	+0.05454	−1
	$\frac{\partial E[I_{1}(T)]/\partial \theta }{\theta^{-1}E[I_{1}(T)]}$	+0.77161	−0.77161	+0.08573	0
	$\frac{\partial \sigma (I_{1}(T))/\partial \theta }{\theta^{-1}\sigma (I_{1}(T))}$	−0.14139	+0.14139	−0.01571	0
	$\frac{\partial E[I_{2}(T)]/\partial \theta }{\theta^{-1}E[I_{2}(T)]}$	−0.92268	+0.92268	−0.10252	0
	$\frac{\partial \sigma (I_{2}(T))/\partial \theta }{\theta^{-1}\sigma (I_{2}(T))}$	−0.14139	+0.14139	−0.01571	0
0.25	$\frac{\partial E[T]/\partial \theta }{\theta^{-1}E[T]}$	−0.69652	−0.30348	−0.00726	−1
	$\frac{\partial \sigma (T)/\partial \theta }{\theta^{-1}\sigma (T)}$	−0.54844	−0.45155	+0.15051	−1
	$\frac{\partial E[I_{1}(T)]/\partial \theta }{\theta^{-1}E[I_{1}(T)]}$	+0.63487	−0.63487	+0.21162	0
	$\frac{\partial \sigma (I_{1}(T))/\partial \theta }{\theta^{-1}\sigma (I_{1}(T))}$	−0.37968	+0.37968	−0.12656	0
	$\frac{\partial E[I_{2}(T)]/\partial \theta }{\theta^{-1}E[I_{2}(T)]}$	−1.03176	+1.03176	−0.34392	0
	$\frac{\partial \sigma (I_{2}(T))/\partial \theta }{\theta^{-1}\sigma (I_{2}(T))}$	−0.37968	+0.37968	−0.12656	0

Note. (Strain 1: antibiotic sensitive; strain 2: antibiotic resistant.)

**Table 3:** Mean and standard deviation of various descriptors in the S I_1_,I_2_ S epidemic model with c=0.25

Descriptor *D*	Expected value *E*[*D*]	Standard deviation ***σ*(*D*)**
*T*	2,481.58612	3,092.40947
$X_{\max}$	16.62473	6.47966
*T*(1)	24.64084	44.53220
*I*_2_(*T*(1))	4.90746	4.58200
$X_{\max}(1)$	6.76017	6.38924
*T*(2)	676.64801	1,105.89870
*I*_1_(*T*(2))	4.51942	4.22930
$X_{\max}(2)$	11.38508	8.79249
*I*_1_(*∞*)	0.81095	2.31470
*I*_2_(*∞*)	11.05264	3.90887

Note. (Strain 1: antibiotic sensitive; strain 2: antibiotic resistant.)

**Table 4:** Elasticities of the descriptors in Table 3 with respect to primary parameters in the S I_1_,I_2_ S epidemic model with c=0.25

Elasticities	***θ* = *β*_1_**	***θ* = *β*_2_**	***θ* = *λ*_1_**	***θ* = *λ*_2_**	***θ* = *γ*_1_**	***θ* = *γ*_2_**
$\frac{\partial E[T]/\partial \theta }{\theta^{-1}E[T]}$	+0.34677	+9.74277	−0.21501	+0.68438	−0.52104	−11.03787
$\frac{\partial \sigma (T)/\partial \theta }{\theta^{-1}\sigma (T)}$	+0.02910	+9.54556	−0.24298	+0.61974	−0.12515	−10.82626
$\frac{\partial E[X_{\max}]/\partial \theta }{\theta^{-1}E[X_{\max}]}$	+0.23125	+0.32876	+0.01805	+0.03876	−0.25994	−0.35689
$\frac{\partial \sigma (X_{\max})/\partial \theta }{\theta^{-1}\sigma (X_{\max})}$	−0.35035	−0.20165	−0.04290	−0.04085	+0.38545	+0.25030
$\frac{\partial E[T(1)]/\partial \theta }{\theta^{-1}E[T(1)]}$	+4.50387	−2.01010	+0.14857	−0.49981	−5.14178	+1.99925
$\frac{\partial \sigma (T(1))/\partial \theta }{\theta^{-1}\sigma (T(1))}$	+4.57286	−2.03753	+0.11877	−0.64958	−5.15409	+2.14957
$\frac{\partial E[I_{2}(T(1))]/\partial \theta }{\theta^{-1}E[I_{2}(T(1))]}$	+0.86287	+1.24945	+0.05036	+0.13550	−1.23667	−1.06153
$\frac{\partial \sigma (I_{2}(T(1)))/\partial \theta }{\theta^{-1}\sigma (I_{2}(T(1)))}$	+0.28166	+0.77701	+0.01704	+0.01477	−0.43910	−0.65139
$\frac{\partial E[X_{\max}(1)]/\partial \theta }{\theta^{-1}E[X_{\max}(1)]}$	+1.69079	−0.57956	+0.07306	−0.08225	−1.60528	+0.50325
$\frac{\partial \sigma (X_{\max}(1))/\partial \theta }{\theta^{-1}\sigma (X_{\max}(1))}$	+1.22316	−0.46291	+0.02971	−0.08852	−1.15905	+0.45761
$\frac{\partial E[T(2)]/\partial \theta }{\theta^{-1}E[T(2)]}$	−4.03536	+9.69147	−0.76852	+0.33206	+4.64273	−10.86238
$\frac{\partial \sigma (T(2))/\partial \theta }{\theta^{-1}\sigma (T(2))}$	−3.73132	+9.10850	−0.75903	+0.27937	+4.35939	−10.25690
$\frac{\partial E[I_{1}(T(2))]/\partial \theta }{\theta^{-1}E[I_{1}(T(2))]}$	+2.29215	+0.40123	+0.23929	+0.03737	−2.36991	−0.60014
$\frac{\partial \sigma (I_{1}(T(2)))/\partial \theta }{\theta^{-1}\sigma (I_{1}(T(2)))}$	+0.98563	+0.09937	+0.01294	+0.01000	−0.93989	−0.16806
$\frac{\partial E[X_{\max}(2)]/\partial \theta }{\theta^{-1}E[X_{\max}(2)]}$	−0.43243	+1.08596	−0.03890	+0.07347	+0.42559	−1.11369
$\frac{\partial \sigma (X_{\max}(2))/\partial \theta }{\theta^{-1}\sigma (X_{\max}(2))}$	−0.11834	+0.29223	−0.01981	+0.00009	+0.14455	−0.29872
$\frac{\partial E[I_{1}(\infty )]/\partial \theta }{\theta^{-1}E[I_{1}(\infty )]}$	+5.62035	−6.43584	+0.98647	−0.55509	−6.60683	+6.99094
$\frac{\partial \sigma (I_{1}(\infty ))/\partial \theta }{\theta^{-1}\sigma (I_{1}(\infty ))}$	+3.76640	−3.79310	+0.45838	−0.37816	−4.22478	+4.17126
$\frac{\partial E[I_{2}(\infty )]/\partial \theta }{\theta^{-1}E[I_{2}(\infty )]}$	−0.43187	+1.31603	−0.08106	+0.06638	+0.51294	−1.38241
$\frac{\partial \sigma (I_{2}(\infty ))/\partial \theta }{\theta^{-1}\sigma (I_{2}(\infty ))}$	+1.25353	−1.70748	+0.18008	−0.19435	−1.43362	+1.90183

Note. (Strain 1: antibiotic sensitive; strain 2: antibiotic resistant.)

In Tables 1 and 2, the interest is in a preliminary scenario with *τ*_1_=*τ*_2_=0.0 (no usage of antibiotics), *γ*=0.0 (no spontaneous recovery), and *μ*=0.0(no arrival or departure of patients during the outbreak), which is readily translated into an *S* *I*_1_,*I*_2_ epidemic model. For practical use, it is worth noting that the derivatives of a predetermined descriptor *D* in Tables 1 and 2 (i.e., expected values and standard deviations of *T*, *I*_1_(*T*) and *I*_2_(*T*)) satisfy

(7) $$\;\frac{\partial D}{\partial c}=-\frac{\beta }{N}\cdot \frac{\partial D}{\partial \beta_{2}},$$

(8) $$\frac{\partial D}{\partial \beta }=\frac{1}{N}\left(\frac{\partial D}{\partial \beta_{1}}+(1-c)\frac{\partial D}{\partial \beta_{2}}\right),$$

because rates *β*_1_ and *β*_2_ depend on the value *c* of fitness cost and the rate *β*; note that Equations (7) and (8) are readily derived from the equalities *∂* *D*/*∂* *c*=(*∂* *D*/*∂* *β*_2_)(*∂* *β*_2_/*∂* *c*) and *∂* *D*/*∂* *β*=(*∂* *D*/*∂* *β*_1_)(*∂* *β*_1_/*∂* *β*)+(*∂* *D*/*∂* *β*_2_)(*∂* *β*_2_/*∂* *β*), respectively. This means that, in implementing Algorithms 1.B and 2.B, we may use *s*=2 parameters (i.e., ***θ***=(*β*_1_,*β*_2_)^*T*^) instead of *s*=4.

In Table 1, we compute the values of the mean length *E*[*T*] of an outbreak and the mean numbers *E*[*I*_1_(*T*)] and *E*[*I*_2_(*T*)] of patients infected by AS and AR bacterial strains, respectively, during the outbreak, together with the corresponding standard deviations. The interest here is in analyzing the impact that small perturbations in the parameters of (*β*_1_,*β*_2_,*c*,*β*) have on these summary statistics, whence in Table 2 we list values of *elasticities* (i.e., (*θ* ^−1^ *D*)^−1^ *∂* *D*/*∂* *θ*) for summary statistics *D* and parameter *θ*. In terms of the sign of *elasticities* (which is identical to the sign of the partial derivative *∂* *D*/*∂* *θ*), the main insights are as follows:

- The mean length of the outbreak *E*[*T*] increases with decreasing values of *β*_1_ and *β*_2_, which are represented by corresponding negative partial derivatives. On the other hand, increasing values of *c* lead to decreasing infectiousness of the AR bacterial strain, which corresponds to longer outbreaks and thus a strictly positive partial derivative *∂* *E*[*T*]/*∂* *c*(that is, it corresponds to longer time until all the patients become infected), whereas increasing global infectiousness *β* leads to decreasing values of *E*[*T*], so that *∂* *E*[*T*]/*∂* *β*<0. These results are explained by noting that no recoveries occur in this model, so that the end of the epidemic spread occurs when all patients are infected.
- The mean number *E*[*I*_1_(*T*)] of infected patients by the AS bacterial strain increases with decreasing values of *β*_2_(because *∂* *E*[*I*_1_(*T*)]/*∂* *β*_2_<0) and with increasing values of *β*_1_ and *c* (because *∂* *E*[*I*_1_(*T*)]/*∂* *β*_1_>0 and *∂* *E*[*I*_1_(*T*)]/*∂* *c*>0), illustrating bacterial strain competition; in a similar manner, analogous comments can be made for the expected number *E*[*I*_2_(*T*)] of patients infected by the strain of AR bacteria.
- Perturbations in the common infection rate *β* do not affect the random variables *I*_1_(*T*) and *I*_2_(*T*) at all, because these perturbations lead to equal relative changes in *β*_1_ and *β*_2_, so that positive and negative effects on these variables are *balanced out*. This is directly related to the fact that the dynamics of the *S* *I*_1_,*I*_2_ epidemic model are governed by the ratio $\beta_{2}^{-1}\beta_{1}$(in this case, becoming (1−*c*)^−1^), and not by the particular magnitudes of *β*_1_ and *β*_2_.
- Stochastic uncertainty, represented by *σ*(*T*), decreases with increasing values of *β*_1_, *β*_2_, and *β*(roughly speaking, the faster infections occur, the less volatile the length of the outbreak is) and with decreasing values of *c*. On the other hand, uncertainty about *I*_1_(*T*), represented by *σ*(*I*_1_(*T*)), increases with *β*_2_(because *∂* *σ*(*I*_1_(*T*))/*∂* *β*_2_>0) and decreases with *β*_1_ and *c*, for similar reasons; note that analogous comments can be made on the strain of AR bacteria in terms of *σ*(*I*_2_(*T*)).

We stress that the comments above refer to the sign of the derivatives in Table 2 and apply regardless of the particular value of *c*∈{0.05,0.1,0.25}. A more detailed comparison between derivatives in absolute terms is carried out by comparing elasticities in Table 2, with the following insights:

- The mean length of the outbreak is more affected by perturbations in *β*_1_ than in *β*_2_, and this difference is more significant with increasing values of *c*. This behavior is directly related to the fact that *β*_2_<*β*_1_, because *c* is strictly positive. In the special case *c*=0, we would expect to obtain a value for the elasticity of *E*[*T*] with respect to *β*_2_(i.e., $Elasticity(E[T];\beta_{2})={\left(\beta_{2}^{-1}E[T]\right)}^{-1}\partial E[T]/\partial \beta_{2}$) equal to its counterpart with respect to *β*_1_(i.e., *E* *l* *a* *s* *t* *i* *c* *i* *t* *y*(*E*[*T*];*β*_1_)). Moreover, the expected length of an outbreak is inversely proportional to *β*, represented by *E* *l* *a* *s* *t* *i* *c* *i* *t* *y*(*E*[*T*];*β*)=−1, which is to be expected because *β* ^−1^=1 day, where 1 day amounts to the unit of time and thus the time unit used for *E*[*T*].
- Some symmetries can be identified; for example, it is seen that
  $$Elasticity(E[I_{1}(T)];\beta_{1})=-Elasticity(E[I_{1}(T)];\beta_{2}),$$
  for any value *c* of fitness cost. This is explained again by the fact that the dynamics in *S* *I*_1_,*I*_2_ epidemic models are governed by the ratio $\beta_{2}^{-1}\beta_{1}$, so that the mean number of patients suffering infection by the AS bacterial strain can increase either by increasing the value of *β*_1_ or decreasing the value of *β*_2_; similar comments apply to the expected number *E*[*I*_2_(*T*)] and standard deviations *σ*(*I*_1_(*T*)) and *σ*(*I*_2_(*T*)).
- In general, the rate *β* represents the most important parameter for the random index *T*, whereas *β*_1_ and *β*_2_ are equally important for the random variables *I*_1_(*T*) and *I*_2_(*T*), regardless of the value of *c*.

We now incorporate discharge and recovery of patients into the model of Lipsitch et al.^31^ by making use of the *S* *I*_1_,*I*_2_ *S* epidemic model with recovery rates *γ*_1_=*γ*+*τ*_1_+*τ*_2_+*μ* and *γ*_2_=*γ*+*τ*_2_+*μ*, and values $\tau_{1}^{-1}=5$ days and $\tau_{2}^{-1}=10$ days, when discharge of patients, who are replaced by susceptible patients, occurs, on average, in 7 days (i.e., *μ* ^−1^=7 days), and spontaneous recovery occurs, on average, in 30 days (i.e., *γ* ^−1^=30 days). These values for *τ*_1_, *τ*_2_, *μ*, and *γ* correspond to realistic selections used by Lipsitch et al. (see figure 2 in the work of Lipsitch et al.^31^), although parameters are known to vary within concrete ranges. For instance, the average duration *μ* ^−1^ of hospital stay and the average time *γ* ^−1^ until the spontaneous clearance of bacterial carriage may vary between 7 and 20 days, and between 30 and 60 days, respectively; see table 1 in the work of Lipsitch et al.^31^ We also select rates *λ*_1_=*N* ^−1^0.1 and *λ*_2_=*N* ^−1^0.1 to represent infections not directly caused by infectious contacts (for example, due to environmental contamination of the hospital ward), but we should point out that these parameters are an addition not considered explicitly in the work of Lipsitch et al.^31^

For the sake of brevity, results in Tables 3 and 4 are related to primary parameters of ***θ***=(*β*_1_,*β*_2_,*λ*_1_,*λ*_2_,*γ*_1_,*γ*_2_)^*T*^ and the choice *c*=0.25; a further discussion on sensitivities and elasticities with respect to secondary parameters (i.e., *c*, *β*, *τ*_1_, *τ*_2_, *μ* and *γ*) can be found online (see Supporting information). We note that long outbreaks obtained in our numerical results (lasting for years; Table 3) are related to the fact that there is an AR bacteria in the hospital ward and that no specific control action is considered in the model. If we focus on the scenario with *c*=0.25, the long global outbreak represented by *E*[*T*]∼2,481 days corresponds to a random overlap of outbreaks corresponding to the AS and AR bacterial strains, until by chance the hospital ward becomes cleared of both strains of bacteria at the same time. The main contribution to this global outbreak length corresponds to long AR bacterial strain outbreaks (with expected length *E*[*T*(2)]∼676 days), overlapping with short AS bacterial strain outbreaks (with expected length *E*[*T*(1)]∼24 days). Moreover, the peak of infection in the hospital ward amounts to $E[X_{\max}]∼16$ infected patients, with peaks of $E[X_{\max}(1)]∼6$ patients infected by the AS bacterial strain, and peaks of $E[X_{\max}(2)]∼11$ patients infected by the AR bacterial strain. Although implementing control measures within the hospital ward would contribute to decrease the values of these summary statistics (more particularly, Lipsitch et al.^31^ considered control strategies such as implementing barrier precautions, improving handwashing compliance levels by health‐care workers, or increasing drug dosage when bacteria are detected in the ward), considering such control actions is out of the scope of this paper, and we focus instead on the local sensitivity analysis for the parameters when no intervention is considered.

In Table 4, we list values of the elasticities of summary statistics with respect to the parameters of (*β*_1_,*β*_2_,*λ*_1_,*λ*_2_,*γ*_1_,*γ*_2_), in the case *c*=0.25. Again, we first focus on the sign of these elasticities (equivalently, partial derivatives). As the reader may observe, the mean length of the outbreak increases with increasing values of *β*_1_, *β*_2_, and *λ*_2_ and with decreasing values of *γ*_1_ and *γ*_2_, as one might expect. However, it is also seen that *∂* *E*[*T*]/*∂* *λ*_1_<0, which suggests that external infections of patients by the strain of AS bacteria act here as a global protection in the hospital ward, reducing the length of the global outbreak. This can be better explained by analyzing scenarios with smaller and larger values of the fitness cost *c*. For example, for *c*=0.1 (results not reported here), we find that *∂* *E*[*T*]/*∂* *β*_1_ and *∂* *E*[*T*]/*∂* *λ*_1_ are strictly negative, so that when the AR bacterial strain is infectious enough, any kind of infection by the strain of AS bacteria acts as a protection measure for the hospital ward in general terms, that is, when analyzing the global outbreak length *E*[*T*]. On the other hand, in the case *c*=0.5, when the fitness cost is large, and as a result, the AR bacterial strain is not so infectious, we find that *∂* *E*[*T*]/*∂* *β*_1_ and *∂* *E*[*T*]/*∂* *λ*_1_ are strictly positive, representing the fact that the protective role of the AS bacterial strain *is not worth it* here, given the low infectiousness of the AR bacterial strain. The scenario in Table 4 (*c*=0.25) should be considered as an intermediate situation, where external infections by AS bacterial strain help to protect the ward, whereas infectious contacts among patients by AS bacterial strain do not play the same protective role. These results suggest that, when considering the implementation of control strategies, special focus should be made on avoiding environmental contamination by the strain of AS bacteria, or on avoiding infectious contacts between patients by the AS bacterial strain (through health‐care workers), depending on the infectiousness of the strain of AR bacteria present in the ward. Other insights from Table 4 are as follows:

- The expected peak $E[X_{\max}]$ of infection increases with increasing values of any rate representing infection and with decreasing values of the recovery rates. On the other hand, expected peaks $E[X_{\max}(1)]$ of infection by the strain of AS bacteria increase with increasing values of *β*_1_, *λ*_1_, and *γ*_2_, and with decreasing values of *β*_2_, *λ*_2_, and *γ*_1_, representing the competition between both strains of bacteria. Similar comments apply not only to AR bacterial strain in terms of $E[X_{\max}(2)]$ but also to the expected steady‐state numbers *E*[*I*_1_(*∞*)] and *E*[*I*_2_(*∞*)] of patients infected by AS and AR bacteria, respectively.
- Derivatives of the standard deviation are difficult to interpret in the *S* *I*_1_,*I*_2_ *S* epidemic model. However, we may note here that, for example, the partial derivatives $\partial \sigma (X_{\max})/\beta_{1}$, $\partial \sigma (X_{\max})/\beta_{2}$, $\partial \sigma (X_{\max})/\lambda_{1}$, and $\partial \sigma (X_{\max})/\lambda_{2}$ are strictly negative, and on the contrary, $\partial \sigma (X_{\max})/\gamma_{1}$ and $\partial \sigma (X_{\max})/\gamma_{2}$ are strictly positive. This means that the peak of the outbreak behaves in a more deterministic way with increasing values of the infection rates, whereas it behaves more stochastically when infection and recovery rates are more balanced, that is, with increasing values of recovery rates in Table 4.

In identifying the most important parameters for each descriptor, an examination of Table 4 reveals the following observations:

- Symmetries identified in the *S* *I*_1_,*I*_2_ epidemic model disappear in the multitype *S* *I* *S* case, because incorporating the recovery of patients in the hospital ward into the *S* *I*_1_,*I*_2_ *S* epidemic model results in a more complex description. In particular, the specific magnitudes of *β*_1_ and *β*_2_ have a significant impact on the descriptors, regardless of maintaining the same value for the ratio $\beta_{2}^{-1}\beta_{1}$.
- When analyzing the expected length *E*[*T*] of the global outbreak, the magnitudes of *β*_2_ and *γ*_2_ are the most relevant ones. This fact is closely related to our comment above where the main contribution to the global outbreak length corresponds to long AR bacterial strain outbreaks. The expected length *E*[*T*(1)] of AS bacterial strain outbreaks is more affected by *β*_1_ and *γ*_1_, whereas *β*_2_ and *γ*_2_ have more impact on the expected length *E*[*T*(2)], as one would expect. However, it is interesting to note that the competition between bacterial strains has a special impact on *E*[*T*(2)], which is represented by relatively large values of *E* *l* *a* *s* *t* *i* *c* *i* *t* *y*(*E*[*T*(2)];*β*_1_) and *E* *l* *a* *s* *t* *i* *c* *i* *t* *y*(*E*[*T*(2)];*γ*_1_). Similar comments directly apply to $E[X_{\max}(1)]$ and $E[X_{\max}(2)]$.
- On the other hand, the global peak $E[X_{\max}]$ of infection, as well as the steady‐state numbers *E*[*I*_1_(*∞*)] and *E*[*I*_2_(*∞*)] of infected patients by strains of AS and AR bacteria, respectively, seem to be approximately equally affected by infection and recovery rates corresponding to both bacterial strains, which result in comparable absolute magnitudes for elasticities of these descriptors with respect to *β*_1_, *β*_2_, *γ*_1_, and *γ*_2_.

## 4 CONCLUSIONS

In this paper, we develop a comprehensive perturbation analysis of finite LD‐QBD processes by computing the partial derivatives of a number of summary statistics with respect to parameters governing the dynamics of the underlying process. This is carried out in an algorithmic fashion that adapts well‐known matrix‐analytic procedures existing in the literature for analyzing this class of Markov chains and by means of using matrix calculus techniques previously applied by Caswell^1^ to absorbing CTMCs. We stress here that this approach could be directly applied to any existing matrix‐analytic algorithmic solution for *skip‐free* Markov chains, which are more elaborated Markov chains than finite LD‐QBD processes. Furthermore, for the finite LD‐QBD process under consideration, perturbation analysis of alternative descriptors allowing for a matrix‐analytic treatment (for example, the number of visits to level *L*(*i*), for integers 1≤*i*≤*K*, before reaching level *L*(0), and numbers of level descents and level ascents before reaching states in *L*(0), among others) could also be developed in a similar manner.

Local sensitivity or perturbation analysis for LD‐QBD processes is specially relevant in epidemic modeling. This is due to the fact that not only the multitype versions of *S* *I* and *S* *I* *S* epidemics can be expressed in terms of LD‐QBD processes but also many other variants—including *S* *I* *R*, *S* *I* *R* *S*, *S* *E* *I* *R* epidemic models—can be represented in this way by conveniently labeling states; see, for example, the works by Artalejo et al.^35^ and Neuts and Li.^36^ In particular, this is possible due to the fact that events such as infections and recoveries in these processes occur one at a time in continuous time. In the special case of *S* *I* *R* epidemic models, the block‐tridiagonal form of **Q** in (1) is linked to a block‐bidiagonal form for transient states (see section 1 in the work of Neuts and Li^36^) when the bivariate process X is defined in terms of the numbers (*S*(*t*),*I*(*t*)) of susceptibles and infectives at time *t*. This block‐bidiagonal form in sections 3 and 4 in the work of Neuts and Li^36^ permits recursive procedures for computing the final size of the epidemic and the maximum size distribution in a similar manner to that in the work of Amador et al.,^37^ where the joint distribution of the maximum number of infectives during an outbreak and the random time to reach this maximum number is also derived in terms of Laplace–Stieltjes transforms. For *S* *I* *R* epidemic models with Markov‐modulated events, we refer the reader to the work of Almaraz and Gómez‐Corral^38^; more concretely, LD‐QBD processes are used in the work of Almaraz and Gómez‐Corral^38^ to derive the probability distributions of the length of an outbreak, the final size of the epidemic, and the number of secondary cases. When these models are used for representing epidemics in reality, statistical estimation techniques, such as Bayesian approaches (for instance, approximate Bayesian computation and Monte Carlo Markov chain methods), are usually implemented in order to estimate parameters of these models from clinical data; see, for example, the work of Kypraios et al.^39^ In the statistical setting, it is therefore important to evaluate the impact that a small perturbation of the underlying parameters may have in the dynamics of the epidemic model, so that one can identify parameters that the model is most sensitive to, allowing for potentially devoting more computational and statistical efforts in estimating those parameter values.

**Figure 3:**
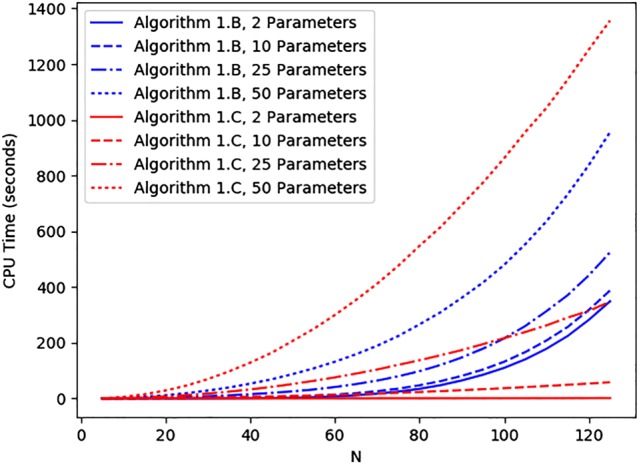
A comparative study between Algorithms 1.B and 1.C

When faced with sensitivities and elasticities, an obvious approach is to evaluate *by direct computation* the partial derivatives *∂* *D*/*∂* *θ*_*r*_ of any descriptor *D* with respect to *θ*_*r*_ for 1≤*r*≤*s*, instead of the differentiation of *D* with respect to the vector ***θ*** of parameters. In the case of first‐passage times and the bacterial transmission model (Section 3.3), Figure 3 shows CPU times for Algorithms 1.B (Section 2.1) and 1.C (Appendix B), where the latter computes, starting at *r*=1, the partial derivatives $\partial E[T_{\left(i,j\right)}^{l}]/\partial \theta_{r}$ for integers 1≤*r*≤*s* and *l*=1, the multitype *S* *I* epidemic model (Section 3.1) and initial state (*i*,*j*)=(*I*_1_+*I*_2_,*I*_2_) with (*I*_1_,*I*_2_)=(1,1). In order to carry out this computational comparison while increasing the number *s* of parameters in ***θ***, we consider *s*−2 additional external sources of type‐1 infection, so that new type‐*k* infections occur with respective rates $i((j+I_{1})\beta_{1}+\sum_{k^{\prime }=1}^{s-2}\lambda_{k^{\prime }})$ if *k*=1, and *i*(*N*−*i*−*j*−*I*_1_)*β*_2_ if *k*=2; this means that, in Figure 3, the number of parameters equals *s* with *s*∈{2,10,25,50}, and the rate $\lambda_{k^{\prime }}$ corresponds to the *k* ^*′*^th external source of type‐1 infection, for 0≤*k* ^*′*^≤*s*−2. It is seen that, for a large number *s* of parameters (*s*=50; Figure 3), Algorithm 1.B behaves better than Algorithm 1.C, whereas the latter performs better than the former if *s* is small (*s*∈{2,10}). This behavior can be explained by using the summary of computational complexity shown in Table 5. To be concrete, Algorithm 1.B (similar to Algorithms 2.B and 3.B) is seen to require an extra effort to construct Jacobian matrices and related matrix operations (for instance, Kronecker products and vectorization of matrices), which are not necessary in Algorithm 1.C. Because the dimension of these matrices depends on the values *s* and *N*, moderate values for the number *s* of parameters (*s*=25) are linked to intermediate situations at which Algorithm 1.B yields better results for small population sizes (*N*<100), but it becomes worse than Algorithm 1.C for larger sizes (*N*>100).

**Table 5:** Computational complexity summary of Algorithms 1.A–B, 2.A–B, and 3.A–B

**Algorithm 1.A**	**Algorithm 1.B**
• It computes the expectations $m_{\left(i,j\right)}^{\left(l\right)}$ of	• It computes partial derivatives $\partial E[T_{\left(i,j\right)}^{l}]/\partial \theta_{r}$,
first‐passage times to level *L*(0), for states	for 1≤*r*≤*s*, states (i,j)∈S\L(0) and
(i,j)∈S\L(0) and integers *l*≥1, from Equation (4).	integers *l*≥1, in a unified manner.
	• It requires the computation of Jacobian matrices
• It involves computing matrices **H**_*i*_, for	$\mathrm{dvec}H_{i}/d\theta^{T}$, for 1≤*i*≤*K*, involving sums,
1≤*i*≤*K*, and their corresponding inverses, with	products, and Kronecker products of matrices
size *M*_*i*_+1.	and vectors, as well as vectorization of matrices,
	with size *M*_*i*_+1. Inverses of matrices previously
	computed in Algorithm 1.A are used.
• Moreover, it consists of two nested loops	• Moreover, it consists of two nested loops
(0≤*p*≤*l*, 1≤*i*≤*K*) involving sums and	(0≤*p*≤*l*, 1≤*i*≤*K*) involving sums, products,
products of matrices of size *M*_*i*_+1.	and Kronecker products of matrices of size
	*M*_*i*_+1, as well as vectorization of these matrices.
**Algorithm 2.A**	**Algorithm 2.B**
• It computes hitting probabilities *p*_(*i*,*j*)_(*n*)	• It computes partial derivatives *∂* *p*_(*i*,*j*)_(*n*)/*∂* *θ*_*r*_,
for states (i,j)∈S\L(0) and phases	for 1≤*r*≤*s*, states (i,j)∈S\L(0) and phases
0≤*n*≤*M*_0_+1.	0≤*n*≤*M*_0_+1, in a unified manner.
	• It consists of three iterations of a loop
• Matrices **H**_*i*_ and corresponding inverses can be	(0≤*i*≤*K*) involving sums, products, and
directly used here once computed in Algorithm1.A.	Kronecker products of matrices and vectors with
	size *M*_*i*_+1, as well as vectorization of these
	matrices. Inverses computed in Algorithm 1.A are used here.
• It consists of three iterations of one loop	
(1≤*i*≤*K*) involving sums and products of	
matrices with size *M*_*i*_+1.	
**Algorithm 3.A**	**Algorithm 3.B**
• It computes stationary probabilities π_(*i*,*j*)_, for	• It computes partial derivatives *∂*π_(*i*,*j*)_/*∂* *θ*_*r*_,
states (i,j)∈S.	for 1≤*r*≤*s* and states (i,j)∈S, in a unified manner.
	• A first loop (1≤*i*≤*K*) constructs Jacobian
	matrices $\mathrm{dvec}B_{i}/\theta^{T}$, involving sums, products,
• It involves computation of inverses $B_{i}^{-1}$, for	and Kronecker products of matrices and vectors
0≤*i*≤*K*, with size *M*_*i*_+1, plus solving a	th size *M*_*i*_+1, as well as vectorization of
system of *M*_*K*_+1 linear equations corresponding	these matrices. It also involves solving a system
to level *L*(*k*).	of linear equations with (*M*_*K*_+1)^2^ equations,
	corresponding to level *L*(*K*) after vectorizing the
	corresponding matrix.
• Moreover, it consists of two subsequent loops	• Moreover, it consists of two subsequent
(0≤*i*≤*K*) involving sums and products of	loops (0≤*i*≤*K*) involving sums, products, and
matrices and vectors with size *M*_*i*_+1.	Kronecker products of matrices and vectors with
	size *M*_*i*_+1, as well as vectorization of these matrices.

Therefore, the use of Algorithms 1.B, 2.B, and 3.B is expected to be more convenient when studying more complex epidemic models, such as those incorporating population heterogeneities at the individual level (considering different individual susceptibilities, infectivities, or recovery periods), leading to LD‐QBD processes defined on networks,^18^, ^40^ these models being specially useful for analyzing epidemic processes in highly heterogeneous environments such as hospital units.^40^, ^41^ The type of local sensitivity analysis carried out here is specially interesting in this type of epidemic processes on networks because the number of parameters in these models grows combinatorially with the number *N* of individuals in the network. For instance, in the case of an *S* *I* *R* epidemic model on a directed network (see the work by López‐García 40) with external sources of infection and *N* individuals, the number of parameters in the model amounts to 2N2+2N, corresponding to 2N2 infectious contact rates, *N* external infection rates, and *N* recovery rates; this means that, for a population of *N*=10 heterogeneous individuals, the number of parameters may be as large as *s*=110.

In a more general setting, Table 6 shows the effect that the number of levels (*K*+1), the numbers of phases per level (*M*_*i*_+1, with 0≤*i*≤*K*), and the number of process parameters (*s*) has on the computational complexity of Algorithms 1.A‐1.B, 2.A‐2.B and 3.A‐3.B. It is observed that Algorithms 1.A and 2.A have computational complexities similar to the computational complexity of the linear level reduction algorithm (Algorithm 3.A) by Gaver et al.,^27^ because the most intensive computational effort lies in these algorithms in the inversion and product of matrices with dimensions *M*_*i*_+1, for 0≤*i*≤*K*. Note that the computational complexity of Algorithms 1.A‐1.B is written by considering a fixed integer *l*.

**Table 6:** Computational complexities of Algorithms 1.A–B, 2.A–B, and 3.A–B

**Algorithm 1.A**	**Algorithm 1.B**
$O\left(\sum_{i=1}^{K}{\left(M_{i}+1\right)}^{3}\right)$	$O\left(max\left\{s\sum_{i=1}^{K-1}{\left(M_{i}+1\right)}^{3}(M_{i+1}+1),\sum_{i=1}^{K}{\left(M_{i}+1\right)}^{5}\right\}\right)$
**Algorithm 2.A**	**Algorithm 2.B**
$O\left(\sum_{i=0}^{K}{\left(M_{i}+1\right)}^{3}\right)$	$O\left(s\sum_{i=0}^{K}{\left(M_{i}+1\right)}^{3}\right)$
**Algorithm 3.A**	**Algorithm 3.B**
$O\left(\sum_{i=0}^{K}{\left(M_{i}+1\right)}^{3}\right)$	$O\left(s\sum_{i=0}^{K}{\left(M_{i}+1\right)}^{4}\right)$

## Supporting information

SupplementaryMaterial_NLA2160.pdf

## APPENDIX A GLOSSARY OF NOTATION AND MATRIX CALCULUS PROPERTIES

### A.1

#### A.1.1

Throughout this paper, vectors and matrices are denoted by bold lowercase and uppercase letters, respectively, with **a** ^*T*^ and **A** ^*T*^ denoting the transpose of the vector **a** and the matrix **A**. We denote the identity matrix of order *h* by **I**_*h*_, the null matrix of dimension *h*×*l* by **0**_*h*×*l*_, and the column vectors of zeros and ones with dimension *h* by **0**_*h*_ and **1**_*h*_, respectively. Given a matrix **A** of dimension *h*×*l* and a column vector **x** with *l* entries, we let *v* *e* *c* **A** be a column vector with *h* *l* entries obtained by *stacking* columns within **A**, and *D*(**x**) denotes the diagonal matrix with **x** within its diagonal.

For a column vector **y** with *h* entries depending on parameters stored in a column vector **x** of order *l*, the derivative of **y** with respect to **x** amounts to the *Jacobian* matrix *d* **y**/*d* **x** ^*T*^ of dimension *h*×*l*, which is given by

$$\frac{dy}{dx^{T}}=\left(\begin{array}{cccc} \frac{dy_{1}}{dx_{1}} & \frac{dy_{1}}{dx_{2}} & \cdots & \frac{dy_{1}}{dx_{l}}\\ \frac{dy_{2}}{dx_{1}} & \frac{dy_{2}}{dx_{2}} & \cdots & \frac{dy_{2}}{dx_{l}}\\ \vdots & \vdots & \ddots & \vdots \\ \frac{dy_{h}}{dx_{1}} & \frac{dy_{h}}{dx_{2}} & \cdots & \frac{dy_{h}}{dx_{l}} \end{array}\right).$$

As a result, derivatives of matrices can be readily derived, translating matrices into vectors by using the *v* *e* *c*(·) operator and by evaluating the resulting Jacobian matrices; more concretely, for a matrix **A** of dimension *m*×*n* with entries *a*_*i**j*_ depending on parameters stored in a column vector **x** of order *l*, the derivative of **A** with respect to **x** is related to the matrix *d* *v* *e* *c* **A**/*d* **x** ^*T*^, which has the form

$$\frac{dvecA}{dx^{T}}=\left(\begin{array}{cccc} \frac{da_{11}}{dx_{1}} & \frac{da_{11}}{dx_{2}} & \cdots & \frac{da_{11}}{dx_{l}}\\ \vdots & \vdots & & \vdots \\ \frac{da_{m1}}{dx_{1}} & \frac{da_{m1}}{dx_{2}} & \cdots & \frac{da_{m1}}{dx_{l}}\\ \frac{da_{12}}{dx_{1}} & \frac{da_{12}}{dx_{2}} & \cdots & \frac{da_{12}}{dx_{l}}\\ \vdots & \vdots & & \vdots \\ \frac{da_{m2}}{dx_{1}} & \frac{da_{m2}}{dx_{2}} & \cdots & \frac{da_{m2}}{dx_{l}}\\ \vdots & \vdots & & \vdots \\ \frac{da_{1n}}{dx_{1}} & \frac{da_{1n}}{dx_{2}} & \cdots & \frac{da_{1n}}{dx_{l}}\\ \vdots & \vdots & & \vdots \\ \frac{da_{mn}}{dx_{1}} & \frac{da_{mn}}{dx_{2}} & \cdots & \frac{da_{mn}}{dx_{l}} \end{array}\right).$$

In Sections 2.1‐2.3, we obtain derivatives of certain properties of X with respect to a vector **w** of parameter values by using four matrix calculus properties, which are as follows:

1. For vectors **y**, **x**, and **z** depending on parameters within **w**, and matrices **W** and **V** with identical numbers of rows, the derivative of **y**=**W** **x**+**V** **z** with respect to **w** is given by
  $$\frac{dy}{dw^{T}}=W\frac{dx}{dw^{T}}+V\frac{dz}{dw^{T}}.$$
2. Let **A** be a square matrix, whose entries depend on parameters within **w**. If the matrix **A** is nonsingular, then the derivative of **A** ^−1^ with respect to **w** can be determined from
  $$\frac{dvec(A^{-1})}{dw^{T}}=-\left({\left(A^{-1}\right)}^{T}⊗A^{-1}\right)\frac{dvecA}{dw^{T}},$$
  where ⊗ represents Kronecker's product.
3. For a column vector **x** with *m* entries depending on parameters within **w**,
  $$\frac{dvecD(\mathrm{x)}}{dw^{T}}=D(vecI_{m})(1_{m}⊗I_{m})\frac{dx}{dw^{T}}.$$
4. For matrices **A**, **B**, and **C** depending on parameters within **w**, it is readily seen that $vec(ABC)=\left(C^{T}⊗A\right)vecB$.

For a general treatment on matrix calculus and its applications, we refer the reader to, among others, the book by Magnus and Neudecker^42^; more concrete results with ecological and demographic applications can be found in the works of Caswell.^1^, ^2^

## APPENDIX B A SIMPLE ALGORITHM

### B.1

#### B.1.1

Starting from *r*=1, Algorithm 1.C allows us to derive partial derivatives of the moments $m_{\left(i,j\right)}^{\left(l\right)}$ of first‐passage times with respect to a single parameter *θ*_*r*_, for integers 1≤*r*≤*s*.

Algorithm 1.C Computation of the partial derivatives $\partial E\left[T_{\left(i,j\right)}^{l}\right]/\partial \theta_{r}$, computed one at a time, for integers *l*≥1 and 1≤*r*≤*s*, and states (i,j)∈S\L(0).

Set *r*=0;
while *r*<*s*, repeat
  *r*=*r*+1;
  Step 1: Set *p*=0;
               for *i*=1,…,*K*, evaluate
  $m_{i}^{\left(p,\theta_{r}\right)}=0_{M_{i}+1}$;
  $b_{i}^{\left(p+1,\theta_{r}\right)}=(p+1)\frac{\partial }{\partial \theta_{r}}\left(\frac{m_{i}^{\left(p\right)}}{\Delta_{i}}\right)$;
  $H_{K}^{\left(\theta_{r}\right)}=-A_{K,K}^{\left(\theta_{r}\right)}$;
               for *i*=*K*−1,…,1, evaluate
  $H_{i}^{\left(\theta_{r}\right)}=-A_{i,i}^{\left(\theta_{r}\right)}-A_{i,i+1}^{\left(\theta_{r}\right)}H_{i+1}^{-1}A_{i+1,i}+A_{i,i+1}H_{i+1}^{-1}H_{i+1}^{\left(\theta_{r}\right)}H_{i+1}^{-1}A_{i+1,i}-A_{i,i+1}H_{i+1}^{-1}A_{i+1,i}^{\left(\theta_{r}\right)}$.
  Step 2: While *p*<*l*, repeat
                              *p*=*p*+1;
  $J_{K}^{\left(p,\theta_{r}\right)}=b_{K}^{\left(p,\theta_{r}\right)}$;
                              for *i*=*K*−1,…,1, evaluate
  $J_{i}^{\left(p,\theta_{r}\right)}=A_{i,i+1}^{\left(\theta_{r}\right)}H_{i+1}^{-1}J_{i+1}^{\left(p\right)}-A_{i,i+1}H_{i+1}^{-1}H_{i+1}^{\left(\theta_{r}\right)}H_{i+1}^{-1}J_{i+1}^{\left(p\right)}+A_{i,i+1}H_{i+1}^{-1}J_{i+1}^{\left(p,\theta_{r}\right)}+b_{i}^{\left(p,\theta_{r}\right)};$
  $m_{1}^{\left(p,\theta_{r}\right)}=-H_{1}^{-1}H_{1}^{\left(\theta_{r}\right)}H_{1}^{-1}J_{1}^{\left(p\right)}+H_{1}^{-1}J_{1}^{\left(p,\theta_{r}\right)}$;
                              for *i*=2,…,*K*, evaluate
  $m_{i}^{\left(p,\theta_{r}\right)}=-H_{i}^{-1}H_{i}^{\left(\theta_{r}\right)}H_{i}^{-1}\left(A_{i,i-1}m_{i-1}^{\left(p\right)}+J_{i}^{\left(p\right)}\right)\;$
  $\;+\;H_{i}^{-1}\left(A_{i,i-1}^{\left(\theta_{r}\right)}m_{i-1}^{\left(p\right)}+A_{i,i-1}m_{i-1}^{\left(p,\theta_{r}\right)}+J_{i}^{\left(p,\theta_{r}\right)}\right)$;
                              for *i*=1,…,*K*, evaluate
  $b_{i}^{\left(p+1,\theta_{r}\right)}=(p+1)\frac{\partial }{\partial \theta_{r}}\left(\frac{m_{i}^{\left(p\right)}}{\Delta_{i}}\right)$.

As the reader may verify, Algorithm 1.C does not use the *v* *e* *c*(·) operator. In Step 2, the column vector $m_{i}^{\left(p,\theta_{r}\right)}$ amounts to the element‐by‐element partial derivative of $m_{i}^{\left(p\right)}$ with respect to parameter *θ*_*r*_, for 1≤*r*≤*s*. Therefore, for the vector of partial derivatives of the element‐by‐element vector division $m_{i}^{\left(p\right)}/\Delta_{i}$, we may express

$${\left(\frac{\partial }{\partial \theta_{r}}\left(\frac{m_{i}^{\left(p\right)}}{\Delta_{i}}\right)\right)}_{j}=\frac{{\left(m_{i}^{\left(p,\theta_{r}\right)}\right)}_{j}{\left(\Delta_{i}\right)}_{j}-{\left(m_{i}^{\left(p\right)}\right)}_{j}{\left(\Delta_{i}^{\left(\theta_{r}\right)}\right)}_{j}}{{\left({\left(\Delta_{i}\right)}_{j}\right)}^{2}}.$$
