# Supplementary material for: Perturbation analysis in finite LD‐QBD processes and applications to epidemic models
Source: Numer Linear Algebra Appl. 2018 Mar 5;25(5):e2160. doi: 10.1002/nla.2160 (PMC6218010; doi:10.1002/nla.2160)
Supplement: Supplementary file 1 — SupplementaryMaterial_NLA2160.pdf [file NLA-25-na-s001.pdf]

Perturbation analysis in finite LD-QBD processes and applications to epidemic models: Supplementary material

A. Gómez-Corral[1 2] and M. López-García[3*]

[1]Instituto de Ciencias Matemáticas CSIC-UAM-UC3M-UCM, Calle Nicolás Cabrera 13-15, 28049 Madrid, Spain
[2] Department of Statistics and Operations Research, School of Mathematics, Complutense University of Madrid, 28040 Madrid, Spain
[3]Department of Applied Mathematics, School of Mathematics, University of Leeds, LS2 9JT Leeds, United Kingdom

SUMMARY

In this manuscript, we present an extended version of [1, Section 3.3], where multi-type SI and SIS epidemic models are applied to the spread of antibiotic-sensitive and antibiotic-resistant bacterial strains in a hospital ward. More concretely, [1, Section 3.3] provides the sensitivities and elasticities of key measures with respect to *primary* parameters, which are linked to the contact and/or recovery rates used in these multi-type epidemic models. The numerical results to be presented here deal with perturbation analysis with respect to those (*secondary*) parameters used by Lipsitch et al. [2] in the underlying deterministic model of bacterial transmission.

1. A MATHEMATICAL MODEL OF BACTERIAL TRANSMISSION

We link the SI_1, I_2 and SI_1, I_2S epidemic models to the deterministic model in [2, Figure A] for the spread of two bacterial strains in a hospital ward. Lipsitch et al. [2] consider an antibiotic-sensitive (AS) bacterial strain and an antibiotic-resistant (AR) bacterial strain -termed *strain 1* and *strain 2*, respectively- spreading among patients, such that the infection by one bacterial strain provides immunity against the other. Because antibiotics are commonly used in hospitals to prevent a wide range of conditions, Lipsitch et al. [2] assume that patients in the ward are routinely provided *antibiotics 1* and *2*, regardless of these patients being or not infected by bacteria; more concretely, antibiotic 1 is only effective against the AS bacterial strain, while antibiotic 2 is effective against both strains of bacteria. The acquisition of resistance by bacteria can lead to some *fitness cost*, amounting to a reduction of the bacterial strain infectiousness due to the corresponding mutation; to represent this fact, Lipsitch et al. [2] consider a common infection rate $\beta = 1.0$ days^{-1}, and set $\beta_1 = \beta$ and $\beta_2 = (1 - c)\beta$ with $c \in (0, 1)$. Spontaneous clearance of sensitive and resistant bacteria occurs at a rate γ, and contributions of antibiotics 1 and 2 to this recovery are represented by rates τ_1 and τ_2. Patients are assumed to be admitted by and discharged from the hospital ward at a common rate μ.

In our numerical experiments (Tables I-VII), we consider a hospital ward with $N = 20$ patients, initial numbers $(I_1, I_2) = (1, 1)$ of infectives, and values $c \in \{0.05, 0.1, 0.25\}$ of fitness cost. It should be pointed out that, unlike the paper [2] where the deterministic model is related to frequencies, we shall from now on consider rates $\beta_1 = N^{-1}\beta$ and $\beta_2 = N^{-1}(1 - c)\beta$, since the random variables in the underlying LD-QBD processes $\mathcal{X}$ ([1, Section 3.1]), and $\mathcal{X}_1$ and $\mathcal{X}_2$ ([1, Section 3.2]) amount to numbers of infectives.

*Correspondence to: Martín López-García, Department of Applied Mathematics, School of Mathematics, University of Leeds, LS2 9JT Leeds, United Kingdom. E-mail: m.lopezgarcia@leeds.ac.uk

Descriptor	$c = 0.05$	$c = 0.1$	$c = 0.25$
$E[T]$	6.19405	6.34476	6.77960
$\sigma(T)$	1.71320	1.75954	1.91860
$E[I_1(T)]$	10.43524	10.89166	12.38137
$\sigma(I_1(T))$	5.46754	5.43652	5.18336
$E[I_2(T)]$	9.56475	9.10833	7.61862
$\sigma(I_2(T))$	5.46754	5.43652	5.18336

Table I. Means and standard deviations of the time T until the end of the epidemic spread, and of the numbers $I_1(T)$ and $I_2(T)$ of type-1 and type-2 infectives when this occurs in the SI_1, I_2 epidemic model. (Strain 1: antibiotic-sensitive; strain 2: antibiotic-resistant.)

In Tables I-III, the interest is in a preliminary scenario with $\tau_1 = \tau_2 = 0.0$ (no usage of antibiotics), $\gamma = 0.0$ (no spontaneous recovery) and $\mu = 0.0$ (no arrival or departure of patients during the outbreak), which is readily translated into an SI_1, I_2 epidemic model. For practical use, it is worth noting that the derivatives of a predetermined descriptor D in Tables I-III (i.e., expected values and standard deviations of T, $I_1(T)$ and $I_2(T)$) satisfy

$$\frac{\partial D}{\partial c} = -\frac{\beta}{N} \cdot \frac{\partial D}{\partial \beta_2}, \quad (1)$$

$$\frac{\partial D}{\partial \beta} = \frac{1}{N}\left(\frac{\partial D}{\partial \beta_1} + (1-c)\frac{\partial D}{\partial \beta_2}\right), \quad (2)$$

since rates β_1 and β_2 depend on the value c of fitness cost and the rate β; note that Eqs. (1)-(2) are readily derived from the equalities $\partial D/\partial c = (\partial D/\partial \beta_2)(\partial \beta_2/\partial c)$ and $\partial D/\partial \beta = (\partial D/\partial \beta_1)(\partial \beta_1/\partial \beta) + (\partial D/\partial \beta_2)(\partial \beta_2/\partial \beta)$, respectively. This means that, in implementing Algorithms 1.B and 2.B in [1], we may use $s = 2$ parameters (i.e., $\boldsymbol{\theta} = (\beta_1, \beta_2)^T$) instead of $s = 4$.

In Table I, we compute the values of the mean length $E[T]$ of an outbreak and the mean numbers $E[I_1(T)]$ and $E[I_2(T)]$ of patients infected by AS and AR bacterial strains, respectively, during the outbreak, together with the corresponding standard deviations. Our interest here is in analyzing the impact that small perturbations in the parameters of $(\beta_1, \beta_2, c, \beta)$ have on these summary statistics. Partial derivatives of interest are given in Table II, and the main insights are as follows:

- The mean length of the outbreak $E[T]$ increases with decreasing values of β_1 and β_2, which is represented by corresponding negative partial derivatives. On the other hand, increasing values of c leads to decreasing infectiousness of the AR bacterial strain, which corresponds to longer outbreaks and thus a strictly positive partial derivative $\partial E[T]/\partial c$ (that is, it corresponds to longer time until all the patients become infected), while increasing global infectiousness β leads to decreasing values of $E[T]$, so that $\partial E[T]/\partial \beta < 0$. These results are explained by noting that no recoveries occur in this model, so that the end of the epidemic spread occurs when all patients are infected.
- The mean number $E[I_1(T)]$ of infected patients by the AS bacterial strain increases with decreasing values of β_2 (since $\partial E[I_1(T)]/\partial \beta_2 < 0$) and with increasing values of β_1 and c (since $\partial E[I_1(T)]/\partial \beta_1 > 0$ and $\partial E[I_1(T)]/\partial c > 0$), illustrating bacterial strain competition; in a similar manner, analogous comments can be made for the expected number $E[I_2(T)]$ of patients infected by the strain of AR bacteria.
- Perturbations in the common infection rate β do not affect the random variables $I_1(T)$ and $I_2(T)$ at all, since these perturbations lead to equal relative changes in β_1 and β_2, so that positive and negative effects on these variables are *balanced out*. This is directly related to the fact that the dynamics of the SI_1, I_2 epidemic model are governed by the ratio $\beta_2^{-1}\beta_1$ (in this case, becoming $(1-c)^{-1}$), and not by the particular magnitudes of β_1 and β_2.
- Stochastic uncertainty –represented by $\sigma(T)$– decreases with increasing values of β_1, β_2 and β (roughly speaking, the fastest infections occur, the less volatile the length of the outbreak is), and with decreasing values of c. On the other hand, uncertainty about $I_1(T)$ –represented

c	Partial derivatives	$\theta = \beta_1$	$\theta = \beta_2$	$\theta = c$	$\theta = \beta$
0.05	$\partial E[T]/\partial\theta$	-66.44025	-60.46403	$+3.02320$	-6.19405
	$\partial\sigma(T)/\partial\theta$	-17.26416	-17.89457	$+0.89472$	-1.71320
	$\partial E[I_1(T)]/\partial\theta$	$+169.42857$	-178.34586	$+8.91729$	0
	$\partial\sigma(I_1(T))/\partial\theta$	-7.54203	$+7.93898$	-0.39694	0
	$\partial E[I_2(T)]/\partial\theta$	-169.42857	$+178.34586$	-8.91729	0
	$\partial\sigma(I_2(T))/\partial\theta$	-7.54203	$+7.93898$	-0.39694	0
0.10	$\partial E[T]/\partial\theta$	-72.87909	-60.01804	$+3.00090$	-6.34476
	$\partial\sigma(T)/\partial\theta$	-17.91517	-19.19523	$+0.95976$	-1.75954
	$\partial E[I_1(T)]/\partial\theta$	$+168.08286$	-186.75874	$+9.33793$	0
	$\partial\sigma(I_1(T))/\partial\theta$	-15.37397	$+17.08219$	-0.85411	0
	$\partial E[I_2(T)]/\partial\theta$	-168.08286	$+186.75874$	-9.33793	0
	$\partial\sigma(I_2(T))/\partial\theta$	-15.37397	$+17.08219$	-0.85411	0
0.25	$\partial E[T]/\partial\theta$	-94.44255	-54.86601	$+2.74330$	-6.77960
	$\partial\sigma(T)/\partial\theta$	-21.04509	-23.10260	$+1.15513$	-1.91860
	$\partial E[I_1(T)]/\partial\theta$	$+157.21245$	-209.61660	$+10.48083$	0
	$\partial\sigma(I_1(T))/\partial\theta$	-39.36084	$+52.48113$	-2.62405	0
	$\partial E[I_2(T)]/\partial\theta$	-157.21245	$+209.61660$	-10.48083	0
	$\partial\sigma(I_2(T))/\partial\theta$	-39.36084	$+52.48113$	-2.62405	0

Table II. Partial derivatives of the descriptors in Table I with respect to various parameters in the SI_1, I_2 epidemic model. (Strain 1: antibiotic-sensitive; strain 2: antibiotic-resistant.)

by $\sigma(I_1(T))$– increases with β_2 (since $\partial\sigma(I_1(T))/\partial\beta_2 > 0$) and decreases with β_1 and c, for similar reasons; note that analogous comments can be made on the strain of AR bacteria in terms of $\sigma(I_2(T))$.

We stress that comments above refer to the sign of the derivatives in Table II and apply regardless of the particular value of $c \in \{0.05, 0.1, 0.25\}$. A more detailed comparison between derivatives in absolute terms can only be carried out after computing their dimensionless counterparts, which are related to *elasticities* (i.e., analyzing $(\theta^{-1}D)^{-1}\partial D/\partial\theta$ instead of $\partial D/\partial\theta$ for any descriptor D and parameter θ). These elasticities are given in Table III, with the following insights:

- The mean length of the outbreak is more affected by perturbations in β_1 than in β_2, and this difference is more significant with increasing values of c. This behavior is directly related to the fact that $\beta_2 < \beta_1$, since c is strictly positive. In fact, we would expect to obtain a value for the elasticity of $E[T]$ with respect to β_2 (i.e., $Elasticity(E[T];\beta_2) = (\beta_2^{-1}E[T])^{-1}\partial E[T]/\partial\beta_2$) equal to its counterpart with respect to β_1 (i.e., $Elasticity(E[T];\beta_1)$), in the special case $c = 0$. Moreover, the expected length of an outbreak is inversely proportional to β –represented by $Elasticity(E[T];\beta) = -1$–, which is to be expected since $\beta^{-1} = 1$ day, where 1 day amounts to the unit of time, and thus the time unit used for $E[T]$.
- Some symmetries can be identified; for example, it is seen that

$$Elasticity(E[I_1(T)];\beta_1) \quad = \quad -Elasticity(E[I_1(T)];\beta_2),$$

  for any value c of fitness cost. This is explained again by the fact that dynamics in SI_1, I_2 epidemic models are governed by the ratio $\beta_2^{-1}\beta_1$, so that the mean number of patients suffering infection by the AS bacterial strain can increase either by increasing the value of β_1 or decreasing the value of β_2; similar comments apply to the expected number $E[I_2(T)]$, and standard deviations $\sigma(I_1(T))$ and $\sigma(I_2(T))$.
- In general, the rate β represents the most important parameter for the random index T, while β_1 and β_2 are equally important for the random variables $I_1(T)$ and $I_2(T)$, regardless of the value of c.

c	Elasticities	$\theta = \beta_1$	$\theta = \beta_2$	$\theta = c$	$\theta = \beta$
0.05	$\frac{\partial E[T]/\partial\theta}{\theta^{-1}E[T]}$	-0.53632	-0.46367	-0.00227	-1
	$\frac{\partial\sigma(T)/\partial\theta}{\theta^{-1}\sigma(T)}$	-0.50385	-0.49614	$+0.02611$	-1
	$\frac{\partial E[I_1(T)]/\partial\theta}{\theta^{-1}E[I_1(T)]}$	$+0.81180$	-0.81180	$+0.04272$	0
	$\frac{\partial\sigma(I_1(T))/\partial\theta}{\theta^{-1}\sigma(I_1(T))}$	-0.06897	$+0.06897$	-0.00363	0
	$\frac{\partial E[I_2(T)]/\partial\theta}{\theta^{-1}E[I_2(T)]}$	-0.88569	$+0.88569$	-0.04661	0
	$\frac{\partial\sigma(I_2(T))/\partial\theta}{\theta^{-1}\sigma(I_2(T))}$	-0.06897	$+0.06897$	-0.00363	0
0.10	$\frac{\partial E[T]/\partial\theta}{\theta^{-1}E[T]}$	-0.57432	-0.42567	-0.00411	-1
	$\frac{\partial\sigma(T)/\partial\theta}{\theta^{-1}\sigma(T)}$	-0.50908	-0.49091	$+0.05454$	-1
	$\frac{\partial E[I_1(T)]/\partial\theta}{\theta^{-1}E[I_1(T)]}$	$+0.77161$	-0.77161	$+0.08573$	0
	$\frac{\partial\sigma(I_1(T))/\partial\theta}{\theta^{-1}\sigma(I_1(T))}$	-0.14139	$+0.14139$	-0.01571	0
	$\frac{\partial E[I_2(T)]/\partial\theta}{\theta^{-1}E[I_2(T)]}$	-0.92268	$+0.92268$	-0.10252	0
	$\frac{\partial\sigma(I_2(T))/\partial\theta}{\theta^{-1}\sigma(I_2(T))}$	-0.14139	$+0.14139$	-0.01571	0
0.25	$\frac{\partial E[T]/\partial\theta}{\theta^{-1}E[T]}$	-0.69652	-0.30348	-0.00726	-1
	$\frac{\partial\sigma(T)/\partial\theta}{\theta^{-1}\sigma(T)}$	-0.54844	-0.45155	$+0.15051$	-1
	$\frac{\partial E[I_1(T)]/\partial\theta}{\theta^{-1}E[I_1(T)]}$	$+0.63487$	-0.63487	$+0.21162$	0
	$\frac{\partial\sigma(I_1(T))/\partial\theta}{\theta^{-1}\sigma(I_1(T))}$	-0.37968	$+0.37968$	-0.12656	0
	$\frac{\partial E[I_2(T)]/\partial\theta}{\theta^{-1}E[I_2(T)]}$	-1.03176	$+1.03176$	-0.34392	0
	$\frac{\partial\sigma(I_2(T))/\partial\theta}{\theta^{-1}\sigma(I_2(T))}$	-0.37968	$+0.37968$	-0.12656	0

Table III. Elasticities of the descriptors in Table I with respect to various parameters in the SI_1, I_2 epidemic model. (Strain 1: antibiotic-sensitive; strain 2: antibiotic-resistant.)

We now incorporate discharge and recovery of patients into the model of Lipsitch et al. [2] by making use of the SI_1, I_2S epidemic model with recovery rates $\gamma_1 = \gamma + \tau_1 + \tau_2 + \mu$ and $\gamma_2 = \gamma + \tau_2 + \mu$, and values $\tau_1^{-1} = 5$ days and $\tau_2^{-1} = 10$ days, when discharge of patients -who are replaced by susceptible patients- occurs in average in 7 days (i.e. $\mu^{-1} = 7$ days), and spontaneous recovery occurs in average in 30 days (i.e., $\gamma^{-1} = 30$ days). These values for τ_1, τ_2, μ and γ correspond to realistic selections used by Lipsitch et al. [2, Figure 2], but parameters are known to vary within concrete ranges. For instance, the average duration μ^{-1} in hospital stay and the average time γ^{-1} until spontaneously clearance of bacterial carriage vary between 7 and 20 days, and between 30 and 60 days, respectively; see [2, Table 1]. We also select rates $\lambda_1 = N^{-1}0.1$ and $\lambda_2 = N^{-1}0.1$ to represent infections not directly caused by infectious contacts (for example, due to environmental contamination of the hospital ward), but we should point out that these parameters are an addition not considered explicitly in [2]. Note that, once the partial derivatives of a certain descriptor D in Tables IV-VII (i.e., expected values and standard deviations of the key measures T, $X_{\max}$, $T(1)$, $X_{\max}(1)$, $T(2)$, $X_{\max}(2)$, $I_1(\infty)$ and $I_2(\infty)$) are obtained with respect to the *primary* parameters of $\boldsymbol{\theta} = (\beta_1, \beta_2, \lambda_1, \lambda_2, \gamma_1, \gamma_2)^T$, we can then obtain derivatives of D with respect to *secondary* parameters (i.e., c, β, τ_1, τ_2, μ and γ) as

$$\begin{aligned} \frac{\partial D}{\partial \tau_1} &= \frac{\partial D}{\partial \gamma_1}, \\ \frac{\partial D}{\partial \tau_2} &= \frac{\partial D}{\partial \gamma_1} + \frac{\partial D}{\partial \gamma_2}, \end{aligned}$$

with $\partial D/\partial c$ and $\partial D/\partial\beta$ verifying Eqs. (1)-(2), and $\partial D/\partial\mu = \partial D/\partial\gamma = \partial D/\partial\tau_2$.

For the sake of brevity, results in Tables IV-VII correspond only to the choice $c = 0.25$. We note that long outbreaks obtained in our numerical results (lasting for years; Table IV) are related to

Descriptor D	Expected value $E[D]$	Standard deviation $\sigma(D)$
T	2481.58612	3092.40947
$X_{\max}$	16.62473	6.47966
$T(1)$	24.64084	44.53220
$I_2(T(1))$	4.90746	4.58200
$X_{\max}(1)$	6.76017	6.38924
$T(2)$	676.64801	1105.89870
$I_1(T(2))$	4.51942	4.22930
$X_{\max}(2)$	11.38508	8.79249
$I_1(\infty)$	0.81095	2.31470
$I_2(\infty)$	11.05264	3.90887

Table IV. Mean and standard deviation of various descriptors in the SI_1, I_2S epidemic model with $c = 0.25$. (Strain 1: antibiotic-sensitive; strain 2: antibiotic-resistant.)

the fact that there is an antibiotic resistant bacteria in the hospital ward and that no specific control action is considered in the model. If we focus on the scenario with $c = 0.25$, the long global outbreak represented by $E[T] \sim 2481$ days corresponds to a random overlap of outbreaks corresponding to the AS and AR bacterial strains, until by chance the hospital ward becomes cleared of both strains of bacteria at the same time. The main contribution to this global outbreak length corresponds to long AR bacterial strain outbreaks (with expected length $E[T(2)] \sim 676$ days), overlapping with short AS bacterial strain outbreaks (with expected length $E[T(1)] \sim 24$ days). Moreover, the peak of infection in the hospital ward amounts to $E[X_{\max}] \sim 16$ infected patients, with peaks of $E[X_{\max}(1)] \sim 6$ patients infected by the AS bacterial strain, and peaks of $E[X_{\max}(2)] \sim 11$ patients infected by the AR bacterial strain. Although implementing control measures within the hospital ward would contribute to decrease the values of these summary statistics (more particularly, Lipsitch et al. [2] consider control strategies such as implementing barrier precautions, improving hand washing compliance levels by healthcare workers, or increasing drug dosage when bacteria is detected in the ward), considering such control actions is out of the scope of this paper, and we focus instead on the local sensitivity analysis for the parameters when no intervention is considered.

In Table V we list values of the partial derivatives of summary statistics with respect to the parameters of $(\beta_1, \beta_2, \lambda_1, \lambda_2, \gamma_1, \gamma_2)$, in the case $c = 0.25$. Again, the focus in this table is on the sign of these derivatives, while analyzing these quantities in absolute terms requires a previous normalization in terms of elasticities. As the reader may observe, the mean length of the outbreak increases with increasing values of β_1, β_2 and λ_2, and with decreasing values of γ_1 and γ_2, as one might expect. However, it is also seen that $\partial E[T]/\partial \lambda_1 < 0$, which suggests that external infections of patients by the strain of AS bacteria act here as a global protection in the hospital ward, reducing the length of the global outbreak. This can be better explained by analyzing scenarios with smaller and larger values of the fitness cost c. For example, for $c = 0.1$ (results not reported here), we find that $\partial E[T]/\partial \beta_1$ and $\partial E[T]/\partial \lambda_1$ are strictly negative, so that when the AR bacterial strain is infectious enough, any kind of infection by the strain of AS bacteria acts as a protection measure for the hospital ward in general terms, that is, when analyzing the global outbreak length $E[T]$. On the other hand, in the case $c = 0.5$ –when the fitness cost is large and, as a result, the AR bacterial strain is not so infectious– we find that $\partial E[T]/\partial \beta_1$ and $\partial E[T]/\partial \lambda_1$ are strictly positive, representing the fact that the protective role of the AS bacterial strain *is not worth it* here, given the low infectiousness of the AR bacterial strain. The scenario in Table V ($c = 0.25$) should be considered as an intermediate situation, where external infections by AS bacterial strain help to protect the ward, while infectious contacts among patients by AS bacterial strain do not play the same protective role. These results suggest that, when considering the implementation of control strategies, more focus on avoiding environmental contamination by the strain of AS bacteria, or on avoiding infectious contacts between patients by AS bacterial strain (through healthcare workers), should be made depending on the suspected infectiousness of the strain of AR bacteria also present in the ward. Other insights from Table V are as follows:

Derivatives	$\theta = \beta_1$	$\theta = \beta_2$	$\theta = \lambda_1$	$\theta = \lambda_2$	$\theta = \gamma_1$	$\theta = \gamma_2$
$\partial E[T]/\partial\theta$	$+17210.826$	$+644733.98$	-106714.99	$+339672.81$	-2715.3157	-99175.879
$\partial\sigma(T)/\partial\theta$	$+1799.8639$	$+787167.59$	-150282.85	$+383302.39$	-812.77832	-121217.96
$\partial E[X_{\max}]/\partial\theta$	$+76.89223$	$+145.75084$	$+60.01742$	$+128.89147$	-9.07532	-21.48238
$\partial\sigma(X_{\max})/\partial\theta$	-45.40336	-34.84429	-55.59569	-52.94084	$+5.24501$	$+5.87235$
$\partial E[T(1)]/\partial\theta$	$+2219.5835$	-1320.8167	$+732.20198$	-2463.1628	-266.06535	$+178.36675$
$\partial\sigma(T(1))/\partial\theta$	$+4072.7944$	-2419.6264	$+1057.8893$	-5785.4950	-481.99831	$+346.59088$
$\partial E[I_2(T(1))]/\partial\theta$	$+84.69066$	$+163.51073$	$+49.43557$	$+132.99655$	-12.74472	-18.86172
$\partial\sigma(I_2(T(1)))/\partial\theta$	$+25.81159$	$+94.94061$	$+15.62153$	$+13.53917$	-4.22516	-10.80658
$\partial E[X_{\max}(1)]/\partial\theta$	$+228.60089$	-104.47893	$+98.78267$	-111.21639	-22.78925	$+12.31799$
$\partial\sigma(X_{\max}(1))/\partial\theta$	$+156.30250$	-78.87120	$+37.97441$	-113.12208	-15.55157	$+10.58616$
$\partial E[T(2)]/\partial\theta$	-54610.453	$+174872.45$	-104004.65	$+44938.345$	$+6597.1446$	-26612.099
$\partial\sigma(T(2))/\partial\theta$	-82529.354	$+268615.62$	-167884.01	$+61791.843$	$+10124.191$	-41069.842
$\partial E[I_1(T(2))]/\partial\theta$	$+207.18418$	$+48.35626$	$+216.29470$	$+33.78191$	-22.49235	-9.82042
$\partial\sigma(I_1(T(2)))/\partial\theta$	$+83.37101$	$+11.20737$	$+10.95274$	$+8.46534$	-8.34774	-2.57358
$\partial E[X_{\max}(2)]/\partial\theta$	-98.46585	$+329.70108$	-88.58496	$+167.30166$	$+10.17548$	-45.90871
$\partial\sigma(X_{\max}(2))/\partial\theta$	-20.81119	$+68.52031$	-34.84539	$+0.16568$	$+2.66899$	-9.50975
$\partial E[I_1(\infty)]/\partial\theta$	$+86.7348$	-133.0172	$+155.7492$	-87.7669	-12.64745	$+18.89745$
$\partial\sigma(I_1(\infty))/\partial\theta$	$+166.22401$	-222.93977	$+205.26701$	-170.67854	-23.11836	$+32.43593$
$\partial E[I_2(\infty)]/\partial\theta$	-89.71266	$+379.86763$	-173.67497	$+143.78424$	$+13.72247$	-53.20738
$\partial\sigma(I_2(\infty))/\partial\theta$	$+92.28649$	-170.18787	$+136.27373$	-148.76542	-13.58669	$+25.33790$

Table V. Partial derivatives of the descriptors in Table IV with respect to primary parameters in the SI_1, I_2S epidemic model with $c = 0.25$. (Strain 1: antibiotic-sensitive; strain 2: antibiotic-resistant.)

- The expected peak $E[X_{\max}]$ of infection increases with increasing values of any rate representing infection, and with decreasing values of the recovery rates. On the other hand, expected peaks $E[X_{\max}(1)]$ of infection by the strain of AS bacteria increase with increasing values of β_1, λ_1 and γ_2, and with decreasing values of β_2, λ_2 and γ_1, representing the competition between both strains of bacteria. Similar comments apply not only to AR bacterial strain in terms of $E[X_{\max}(2)]$, but also to the expected steady-state numbers $E[I_1(\infty)]$ and $E[I_2(\infty)]$ of patients infected by AS and AR bacteria, respectively.
- Derivatives of the standard deviation are difficult to interpret in the SI_1, I_2S epidemic model. However, we may note here that, for example, the partial derivatives $\partial\sigma(X_{\max})/\beta_1$, $\partial\sigma(X_{\max})/\beta_2$, $\partial\sigma(X_{\max})/\lambda_1$ and $\partial\sigma(X_{\max})/\lambda_2$ are strictly negative and, on the contrary, $\partial\sigma(X_{\max})/\gamma_1$ and $\partial\sigma(X_{\max})/\gamma_2$ are strictly positive. This means that the peak of the outbreak behaves in a more deterministic way with increasing values of the infection rates, while it behaves more stochastically when infection and recovery rates are more balanced, that is, with increasing values of recovery rates in Table V.

In order to identify the most important parameters for each descriptor, we list in Table VI the elasticities of summary statistics with respect to the parameters. An examination of Table VI reveals the following observations:

- Symmetries identified in the SI_1, I_2 epidemic model disappear in the multi-type SIS case, since incorporating recovery of patients in the hospital ward into the SI_1, I_2S epidemic model results in a more complex description. In particular, the specific magnitudes of β_1 and β_2 have a significant impact on the descriptors, regardless of maintaining the same value for the ratio $\beta_2^{-1}\beta_1$.
- When analyzing the expected length $E[T]$ of the global outbreak, the magnitudes of β_2 and γ_2 are the most relevant ones. This fact is closely related to our comment above where the main contribution to the global outbreak length corresponds to long AR bacterial strain outbreaks. The expected length $E[T(1)]$ of AS bacterial strain outbreaks is more affected by β_1 and γ_1, while β_2 and γ_2 have more impact on the expected length $E[T(2)]$, as

Elasticities	$\theta = \beta_1$	$\theta = \beta_2$	$\theta = \lambda_1$	$\theta = \lambda_2$	$\theta = \gamma_1$	$\theta = \gamma_2$
$\frac{\partial E[T]/\partial\theta}{\theta^{-1}E[T]}$	$+0.34677$	$+9.74277$	-0.21501	$+0.68438$	-0.52104	-11.03787
$\frac{\partial\sigma(T)/\partial\theta}{\theta^{-1}\sigma(T)}$	$+0.02910$	$+9.54556$	-0.24298	$+0.61974$	-0.12515	-10.82626
$\frac{\partial E[X_{\max}]/\partial\theta}{\theta^{-1}E[X_{\max}]}$	$+0.23125$	$+0.32876$	$+0.01805$	$+0.03876$	-0.25994	-0.35689
$\frac{\partial\sigma(X_{\max})/\partial\theta}{\theta^{-1}\sigma(X_{\max})}$	-0.35035	-0.20165	-0.04290	-0.04085	$+0.38545$	$+0.25030$
$\frac{\partial E[T(1)]/\partial\theta}{\theta^{-1}E[T(1)]}$	$+4.50387$	-2.01010	$+0.14857$	-0.49981	-5.14178	$+1.99925$
$\frac{\partial\sigma(T(1))/\partial\theta}{\theta^{-1}\sigma(T(1))}$	$+4.57286$	-2.03753	$+0.11877$	-0.64958	-5.15409	$+2.14957$
$\frac{\partial E[I_2(T(1))]/\partial\theta}{\theta^{-1}E[I_2(T(1))]}$	$+0.86287$	$+1.24945$	$+0.05036$	$+0.13550$	-1.23667	-1.06153
$\frac{\partial\sigma(I_2(T(1)))/\partial\theta}{\theta^{-1}\sigma(I_2(T(1)))}$	$+0.28166$	$+0.77701$	$+0.01704$	$+0.01477$	-0.43910	-0.65139
$\frac{\partial E[X_{\max}(1)]/\partial\theta}{\theta^{-1}E[X_{\max}(1)]}$	$+1.69079$	-0.57956	$+0.07306$	-0.08225	-1.60528	$+0.50325$
$\frac{\partial\sigma(X_{\max}(1))/\partial\theta}{\theta^{-1}\sigma(X_{\max}(1))}$	$+1.22316$	-0.46291	$+0.02971$	-0.08852	-1.15905	$+0.45761$
$\frac{\partial E[T(2)]/\partial\theta}{\theta^{-1}E[T(2)]}$	-4.03536	$+9.69147$	-0.76852	$+0.33206$	$+4.64273$	-10.86238
$\frac{\partial\sigma(T(2))/\partial\theta}{\theta^{-1}\sigma(T(2))}$	-3.73132	$+9.10850$	-0.75903	$+0.27937$	$+4.35939$	-10.25690
$\frac{\partial E[I_1(T(2))]/\partial\theta}{\theta^{-1}E[I_1(T(2))]}$	$+2.29215$	$+0.40123$	$+0.23929$	$+0.03737$	-2.36991	-0.60014
$\frac{\partial\sigma(I_1(T(2)))/\partial\theta}{\theta^{-1}\sigma(I_1(T(2)))}$	$+0.98563$	$+0.09937$	$+0.01294$	$+0.01000$	-0.93989	-0.16806
$\frac{\partial E[X_{\max}(2)]/\partial\theta}{\theta^{-1}E[X_{\max}(2)]}$	-0.43243	$+1.08596$	-0.03890	$+0.07347$	$+0.42559$	-1.11369
$\frac{\partial\sigma(X_{\max}(2))/\partial\theta}{\theta^{-1}\sigma(X_{\max}(2))}$	-0.11834	$+0.29223$	-0.01981	$+0.00009$	$+0.14455$	-0.29872
$\frac{\partial E[I_1(\infty)]/\partial\theta}{\theta^{-1}E[I_1(\infty)]}$	$+5.62035$	-6.43584	$+0.98647$	-0.55509	-6.60683	$+6.99094$
$\frac{\partial\sigma(I_1(\infty))/\partial\theta}{\theta^{-1}\sigma(I_1(\infty))}$	$+3.76640$	-3.79310	$+0.45838$	-0.37816	-4.22478	$+4.17126$
$\frac{\partial E[I_2(\infty)]/\partial\theta}{\theta^{-1}E[I_2(\infty)]}$	-0.43187	$+1.31603$	-0.08106	$+0.06638$	$+0.51294$	-1.38241
$\frac{\partial\sigma(I_2(\infty))/\partial\theta}{\theta^{-1}\sigma(I_2(\infty))}$	$+1.25353$	-1.70748	$+0.18008$	-0.19435	-1.43362	$+1.90183$

Table VI. Elasticities of the descriptors in Table IV with respect to primary parameters in the SI_1, I_2S epidemic model with $c = 0.25$. (Strain 1: antibiotic-sensitive; strain 2: antibiotic-resistant.)

one would expect. However, it is interesting to note that the competition between bacterial strains has a special impact on $E[T(2)]$, which is represented by relatively large values of $Elasticity(E[T(2)];\beta_1)$ and $Elasticity(E[T(2)];\gamma_1)$. Similar comments directly apply to $E[X_{\max}(1)]$ and $E[X_{\max}(2)]$.

- On the other hand, the global peak $E[X_{\max}]$ of infection, as well as the steady-state numbers $E[I_1(\infty)]$ and $E[I_2(\infty)]$ of infected patients by strains of AS and AR bacteria, respectively, seem to be approximately equally affected by infection and recovery rates corresponding to both bacterial strains, which results in comparable absolute magnitudes for elasticities of these descriptors with respect to β_1, β_2, γ_1 and γ_2.

Finally, we list in Table VII the elasticities of descriptors with respect to the parameters of $(\tau_1, \tau_2, \gamma, \mu, c, \beta)$, with the following insights:

- The most relevant parameters correspond to μ, c and β for most of the summary statistics. This means that the discharge of patients (who might be carrying the bacteria), the infectiousness of the bacterial species (represented by β) and the fitness cost c of the antibiotic-bacterial strain are the most important factors affecting the dynamics of these infections. Since discharge of patients carrying the bacteria implies clear ethical concerns not addressed in Ref. [2], and parameters c and β correspond to factors that can not be controlled by policy-makers in the hospital ward, these elasticities are less helpful in terms of analyzing the efficacy of potential control strategies. Policy-making related parameters in this model correspond to the usage of antibiotics 1 and 2.

Elasticities	$\theta = \tau_1$	$\theta = \tau_2$	$\theta = \gamma$	$\theta = \mu$	$\theta = c$	$\theta = \beta$
$\frac{\partial E[T]/\partial\theta}{\theta^{-1}E[T]}$	-0.21883	-4.10589	-1.36863	-5.86555	-3.24759	$+10.08954$
$\frac{\partial \sigma(T)/\partial\theta}{\theta^{-1}\sigma(T)}$	-0.05256	-3.94613	-1.31537	-5.63734	-3.18185	$+9.57466$
$\frac{\partial E[X_{\max}]/\partial\theta}{\theta^{-1}E[X_{\max}]}$	-0.10917	-0.18380	-0.06127	-0.26258	-0.10958	$+0.56002$
$\frac{\partial \sigma(X_{\max})/\partial\theta}{\theta^{-1}\sigma(X_{\max})}$	$+0.16189$	$+0.17157$	$+0.05719$	$+0.24510$	$+0.06721$	-0.55200
$\frac{\partial E[T(1)]/\partial\theta}{\theta^{-1}E[T(1)]}$	-2.15954	-0.35590	-0.11863	-0.50843	$+0.67003$	$+2.49376$
$\frac{\partial \sigma(T(1))/\partial\theta}{\theta^{-1}\sigma(T(1))}$	-2.16471	-0.30406	-0.10135	-0.43438	$+0.67917$	$+2.53532$
$\frac{\partial E[I_2(T(1))]/\partial\theta}{\theta^{-1}E[I_2(T(1))]}$	-0.51940	-0.64404	-0.21468	-0.92006	-0.41648	$+2.11233$
$\frac{\partial \sigma(I_2(T(1)))/\partial\theta}{\theta^{-1}\sigma(I_2(T(1)))}$	-0.18442	-0.32806	-0.10935	-0.46865	-0.25900	$+1.05867$
$\frac{\partial E[X_{\max}(1)]/\partial\theta}{\theta^{-1}E[X_{\max}(1)]}$	-0.67422	-0.15489	-0.05163	-0.22128	$+0.19318$	$+1.11122$
$\frac{\partial \sigma(X_{\max}(1))/\partial\theta}{\theta^{-1}\sigma(X^{max}(1))}$	-0.48680	-0.07771	-0.02590	-0.11102	$+0.15430$	$+0.76025$
$\frac{\partial E[T(2)]/\partial\theta}{\theta^{-1}E[T(2)]}$	$+1.94994$	-2.95795	-0.98598	-4.22565	-3.23049	$+5.65610$
$\frac{\partial \sigma(T(2))/\partial\theta}{\theta^{-1}\sigma(T(2))}$	$+1.83094$	-2.79823	-0.93274	-3.99747	-3.03616	$+5.37718$
$\frac{\partial E[I_1(T(2))]/\partial\theta}{\theta^{-1}E[I_1(T(2))]}$	-0.99536	-0.71497	-0.23832	-1.02139	-0.13374	$+2.69338$
$\frac{\partial \sigma(I_1(T(2)))/\partial\theta}{\theta^{-1}\sigma(I_1(T(2)))}$	-0.39475	-0.25823	-0.08607	-0.36890	-0.03312	$+1.08500$
$\frac{\partial E[X_{\max}(2)]/\partial\theta}{\theta^{-1}E[X_{\max}(2)]}$	$+0.17875$	-0.31386	-0.10462	-0.44837	-0.36198	$+0.65353$
$\frac{\partial \sigma(X_{\max}(2))/\partial\theta}{\theta^{-1}\sigma(X_{\max}(2))}$	$+0.06071$	-0.07780	-0.02593	-0.11114	-0.09741	$+0.17389$
$\frac{\partial E[I_1(\infty)]/\partial\theta}{\theta^{-1}E[I_1(\infty)]}$	-2.77487	$+1.14376$	$+0.38125$	$+1.63395$	$+2.14528$	-0.81548
$\frac{\partial \sigma(I_1(\infty))/\partial\theta}{\theta^{-1}\sigma(I_1(\infty))}$	-1.77441	$+0.62307$	$+0.20769$	$+0.89011$	$+1.26436$	-0.02669
$\frac{\partial E[I_2(\infty)]/\partial\theta}{\theta^{-1}E[I_2(\infty)]}$	$+0.21543$	-0.39281	-0.13093	-0.56115	-0.43867	$+0.88415$
$\frac{\partial \sigma(I_2(\infty))/\partial\theta}{\theta^{-1}\sigma(I_2(\infty))}$	-0.60212	$+0.38753$	$+0.12917$	$+0.55362$	$+0.56916$	-0.45394

Table VII. Elasticities of the descriptors in Table IV with respect to secondary parameters in the SI_1, I_2S epidemic model with $c = 0.25$. (Strain 1: antibiotic-sensitive; strain 2: antibiotic-resistant.)

- The usage of antibiotic 2, which is effective against both strains of bacteria, is specially important for reducing the length of the outbreak (i.e., large values of $Elasticity(E[T]; \tau_2) < 0$ in absolute terms), since it is important in order to reduce the length of the outbreak corresponding to the AR bacterial strain (i.e., large values of $Elasticity(E[T(2)]; \tau_2) < 0$ in absolute terms). It also plays a role in reducing the peak of the general outbreak (expressed in terms of $Elasticity(E[X_{\max}]; \tau_2)$) of the AR bacterial strain outbreak (expressed in terms of $Elasticity(E[X_{\max}(2)]; \tau_2)$).
- The usage of antibiotic 1, which is only effective against the strain of AS bacteria, has an obvious positive impact for controlling AS bacterial strain outbreaks, but it has however a negative impact for controlling AR bacterial strain outbreaks. This is related to the fact that antibiotic 1 has no direct impact on the recovery of patients infected by AR bacterial strain, but at the same time it helps to remove its direct competitor (i.e., the AS bacterial strain), leading for example to strictly positive magnitudes for $Elasticity(E[T(2)]; \tau_1)$. These positive and negative effects seem however to be compensated from a general perspective, leading to elasticities of $E[T]$ and $E[X_{\max}]$ with respect to τ_1 near zero.

REFERENCES

1. Gómez-Corral A, López-García M. Perturbation analysis in finite LD-QBD processes and applications to epidemic models. *Numerical Linear Algebra With Applications* 2017.
2. Lipsitch M, Bergstrom CT, Levin BR. The epidemiology of antibiotic resistance in hospitals: Paradoxes and prescriptions. *Proceedings of the National Academy of Sciences of the U.S.A.* 2000; **97:**1938-1943.
